# A Cryptic Pocket Allosterically Modulates Oligosaccharide Binding to DC-SIGN

**DOI:** 10.1021/jacsau.5c01465

**Published:** 2026-01-20

**Authors:** Jonathan Lefèbre, Maurice Besch, Marcelo Daniel Gamarra, Jan-Oliver Kapp-Joswig, Annika Balke, Stevan Aleksić, Henry Flatau, Gregor Suchy, Elena Georgieva, Patrick Scheerer, Bettina G. Keller, Carlos Pablo Modenutti, Christoph Rademacher

**Affiliations:** 1 Department of Pharmaceutical Sciences, 27258 University of Vienna, Josef-Holaubek-Platz 2, Vienna 1090, Austria; 2 Vienna Doctoral School of Pharmaceutical, Nutritional and Sport Sciences, 27258 University of Vienna, Josef-Holaubek-Platz 2, Vienna 1090, Austria; 3 Department of Microbiology, Immunology and Genetics, University of Vienna, Max F. Perutz Laboratories, Dr. Bohr Gasse 9, Vienna 1030, Austria; 4 Departamento de Química Biológica, Facultad de Ciencias Exactas y Naturales, Universidad de Buenos Aires (FCEyN-UBA), Ciudad de Buenos Aires C1428EGA, Argentina; 5 Instituto de Química Biológica de la Facultad de Ciencias Exactas y Naturales (IQUIBICEN) CONICET, Pabellón 2 de Ciudad Universitaria, Ciudad de Buenos Aires C1428EHA, Argentina; 6 Department of Biology, Chemistry, Pharmacy, Freie Universität Berlin, Arnimallee 22, Berlin 14195, Germany; 7 Institute of Medical Physics and Biophysics, Group Structural Biology of Cellular Signaling, Charité - Universitätsmedizin Berlin, corporate member of Freie Universität Berlin, Humboldt-Universität zu Berlin, Berlin 10117, Germany; 8 Istituto di Biologia e Biotecnologia Agraria (IBBA), Consiglio Nazionale della Ricerca (CNR), Via Alfonso Corti nr. 12, Milano 20133, Italia

**Keywords:** C-type lectin, DC-SIGN, allostery, cryptic pocket, NMR

## Abstract

DC-SIGN is a C-type lectin receptor expressed on antigen-presenting cells that is crucial for pathogen recognition and immune modulation. Here, we identify and characterize a previously unrecognized cryptic allosteric pocket in DC-SIGN using molecular dynamics simulations, NMR spectroscopy, cryogenic electron microscopy, and biochemical assays. Rotation of the gatekeeper residue M270 exposes the pocket whose occupancy modulates glycan binding. Mutations M270F and T314A mimic the occupied and unoccupied states of this pocket, respectively, shifting the conformational equilibrium of α-helix 2 and altering the oligosaccharide affinity via the extended carbohydrate binding site. While Ca^2+^ coordination at the canonical binding site remains unaffected, our data reveal a complex interplay between the Ca^2+^ binding sites and the canonical and extended glycan binding surfaces. These findings uncover a hierarchical allosteric mechanism that enables selective tuning of glycan affinity and suggest the cryptic pocket as a novel target for drug discovery in C-type lectins.

## Graphical abstract

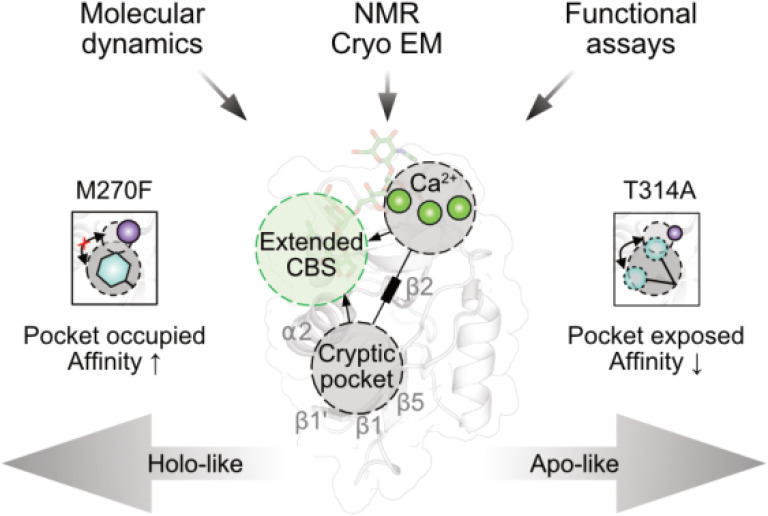

## Introduction

Dendritic cell-specific intercellular adhesion molecule-3-grabbing nonintegrin (DC-SIGN) is a Ca^2+^-dependent glycan binding C-type lectin receptor (CLR) expressed on antigen-presenting cells, including dendritic cells and macrophages.^1,2^ It plays a central role in bridging innate and adaptive immunity by discriminating self from nonself, facilitating pathogen recognition, cellular adhesion, and immunological synapse formation.^3–9^ The receptor recognizes a broad range of carbohydrate structures, most prominently high-mannose and fucose-containing glycans, such as Lewis X.^10–12^ These include glycans from bacterial and fungal pathogens, as well as viral glycoproteins exploited, for example, by HIV-1 and SARS-CoV-2 for cellular entry and dissemination.^13–19^ DC-SIGN also recognizes self-antigens on endogenous glycoproteins, highlighting the diversity of ligands and outcomes ranging from pathogen uptake and immune activation to tolerance.^1,20,21^ This biological versatility makes it an attractive target for antiviral and immunomodulatory strategies as well as targeted delivery.^9,22–24^

Critical to the function of DC-SIGN is its ability to recognize a diverse repertoire of glycan structures through its carbohydrate recognition domain (CRD). The DC-SIGN CRD binds three Ca^2+^ ions at its long loop region, one of which is coordinated by the conserved Glu-Pro-Asn (EPN) motif, essential for recognizing mannose and fucose-containing glycans at the canonical carbohydrate binding site (CBS).^25,26^ To achieve functional binding, DC-SIGN engages not only this canonical binding site but also an extended CBS formed by β-strands β2–4, α-helix α2, and residues of the α2−β2 connecting loop (Figure 1A).^25^ This extended site supports recognition of oligosaccharides in multiple binding modes and contributes to secondary contacts that enhance binding affinity and specificity.^27^

**Figure 1:**
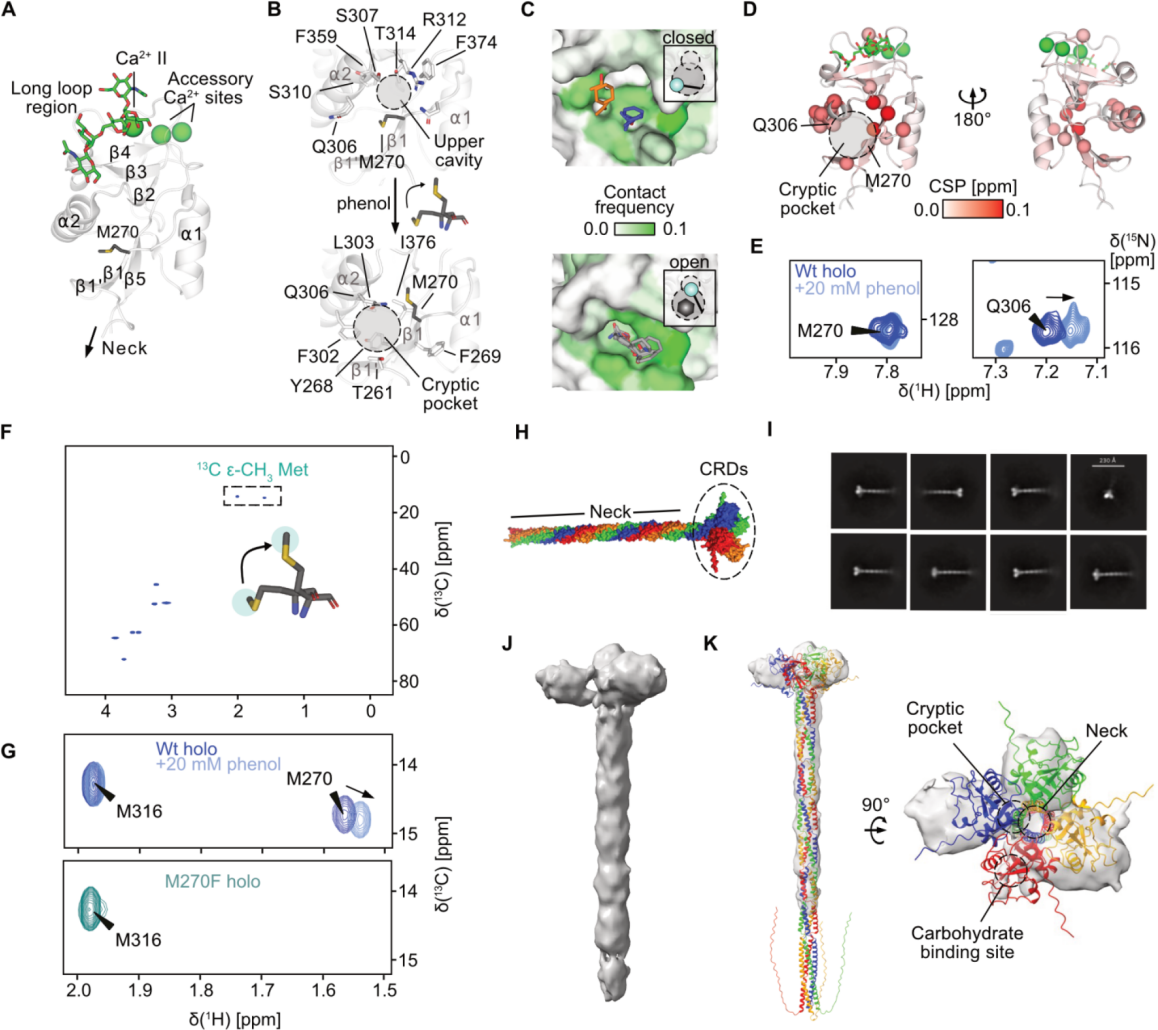
DC-SIGN harbors a cryptic secondary site. (A) X-ray structure of DC-SIGN CRD with GlcNAc_2_Man_3_ (PDB 1K9I) showing the canonical Ca^2+^-dependent CBS in the long loop and the extended CBS formed by β2–4, α2, and the α2−β2 loop.^26^ A previously identified secondary site between α2 and β1′, 1 and 5 is centered by residue M270.^28^ (B) Mixed-solvent MD simulations of the CRD with phenol reveal M270 rotates into an upper cavity (Q306, S307, S310, R312, T314, F374, and F359), opening a hydrophobic cryptic pocket (T261, Y268, F269, M270, F302, L303, and I376). (C) FTMap analysis of the closed (top) and open (bottom) states suggests higher druggability upon cryptic pocket opening. Solvent probe clusters are shown as orange and blue sticks and gray sticks for the closed and open state, respectively. Surface is colored in green according to probe contact frequencies.^29^ (D) CSPs observed in ^1^H–^15^N HSQC NMR experiments with 20 mM phenol and holo ^15^N-labeled CRD validate the binding site of phenol at the cryptic pocket. Cα atoms of residues experiencing a CSP > 0.025 ppm are shown as spheres. (E) Resonances of residues M270 and Q306 are shown. Minimal perturbation of M270 suggests limited changes in the amide backbone of the residue. (F) ^13^C Met labeling provides ^1^H–^13^C HSQC NMR spectra of the methionine side chains of residues M316 and M270. (G) Addition of phenol opens the cryptic pocket, as indicated by a CSP induced in the M270 resonance (top). Assignment of the methionine resonances was done using a M270F mutant (bottom). (H) Alphafold3 model of the DC-SIGN ECD tetramer (Figure S4).^30^ Adapted from ref 31. Available under a CC-BY NC 4.0 license. Copyright 2025, Lefèbre et al. (I) Representative 2D class averages of DC-SIGN ECD particles, showing predominantly side and intermediate views, with top views being underrepresented. Scale bar: 230 Å. (J) Cryo-EM map of DC-SIGN ECD showing the expected tetrameric topology, with three of the four CRDs visibly resolved. (K) Rigid body docking of the cryo-EM map to the Alphafold3 model reveals a different arrangement of the CRDs, resulting from high flexibility of the CRD-neck junction.^32^ The relative positions of the cryptic pocket, the CBS and the CRD-neck junction are highlighted with dotted circles. The cryo-EM map is deposited in the EMDB under accession EMD-56237.

Under physiological conditions the DC-SIGN extracellular domain (ECD) forms tetramers via its neck region allowing for multivalent recognition of glycans, further amplifying ligand binding by avidity effects, eventually leading to endocytosis and immune signaling.^33–35^ The cellular response is highly context-dependent and can vary significantly with antigen structure and the glycan involved.^36–39^ Accordingly, while this multilevel architecture enables functional plasticity, it also raises questions about how glycan binding at the CRD translates into cellular responses, particularly in light of the structure–function relationship that governs signaling and endocytic activity of other CLRs, such as langerin, MGL and CLEC5A.^40–43^ Understanding conformational dynamics and regulatory mechanisms may enable the rational design of compounds that inhibit, activate, or fine-tune the activity of DC-SIGN in the context of immunity and infection.

Despite its therapeutic potential, designing small molecules that directly target the CBS of DC-SIGN with high selectivity and affinity remains challenging. The site is shallow, polar, and binding usually involves a large entropic penalty due to loop flexibility and solvent exposure, resulting in low-affinity and high promiscuity of DC-SIGN-carbohydrate interactions.^44,45^ An emerging strategy to circumvent these limitations involves targeting secondary sites that are distinct from the CBS and potentially provide increased selectivity, functional specificity, and new opportunities for therapeutic intervention.^46^ In DC-SIGN, fragment screening and computational studies have revealed the presence of several secondary sites, including one distal site between α2, β1′, β1, and β5 that is centered by residue M270 (Figure 1A).^47–53^ The same site was later exploited to target DC-SIGN-expressing cells using heteromultivalent liposomes and was suggested to allosterically modulate Ca^2+^-dependent carbohydrate binding by an unknown mechanism.^28^

In this study, we combined molecular dynamics (MD) simulations, nuclear magnetic resonance (NMR) spectroscopy, and biochemical assays to identify and characterize a previously unrecognized druggable pocket in DC-SIGN, accessible only upon conformational rearrangement. We show that the rotational shift of the “gatekeeper” residue M270 exposes this cryptic pocket whose occupancy modulates glycan binding allosterically at the CBS. Introducing M270F and T314A mutations mimics the occupied and the unoccupied states of the pocket, respectively, and shifts the conformational equilibrium of α2, thereby tuning oligosaccharide affinity via the extended CBS. We excluded a direct effect on the Ca^2+^ cage but found evidence for a more complex interplay of the Ca^2+^ sites, the canonical and the extended CBS. Together, these results reveal a hierarchical allosteric mechanism that primes DC-SIGN for selective glycan engagement and opens new avenues for drug discovery targeted at CLRs.

## Results

### Gatekeeper Residue M270 Controls Cryptic Pocket Opening

Previous fragment screening of DC-SIGN identified hits interacting with secondary sites, while *in silico* analysis indicated low druggability.^28,49,50^ To expand our knowledge on the local dynamics and druggability of secondary sites in DC-SIGN, we conducted mixed-solvent MD simulations of the CRD with and without phenol to explore the dynamics of these sites.^54^ Simulations in 5% v/v water/phenol mixtures identified hotspots matching previously described secondary sites (Figure S1A).^49^ While backbone conformational changes were minimal, a hotspot between α2 and β5 was only revealed upon rotation of the thioether side chain of residue M270 into a small upper cavity at the C-terminal end of α2 (Figure 1B). This motion expelled water and exposed a hydrophobic cryptic pocket with increased druggability, as predicted by FTMap (Figure 1C and Figure S1B).^29^ Without phenol, the open state was still populated (∼34%), indicating an equilibrium between the open and closed states in the absence of phenol (Figure S1C,D).

To experimentally verify the interaction of small organic molecules with the cryptic pocket, we titrated phenol at high excess to uniformly ^15^N-labeled DC-SIGN CRD in ^1^H–^15^N heteronuclear single quantum coherence (HSQC) NMR experiments. We observed strong chemical shift perturbations (CSPs) in residues of the predicted cryptic pocket as well as the upper cavity, to which the M270 side chain rotates (Figure 1D). In support of a direct interaction with the pocket, measurements with a previously established M270F mutant that blocks secondary site binding showed significantly less CSPs around that site (Figure S2).^28^

Although ^1^H–^15^N HSQC NMR allowed us to infer direct binding to the site around M270, CSPs in the M270 resonance were small (Figure 1E). Since the mechanism of opening of the pocket largely depends on rotation of the methionine side chain instead of backbone movements, we expressed ^13^C ε- methyl methionine (^13^C Met)-labeled CRD and assigned the corresponding ^1^H–^13^C resonance of M270 by comparison with spectra of the M270F mutant (Figure 1F,G and Figure S3). In line with our observations from simulations, the addition of phenol induced a CSP in the M270 resonance, indicative of pocket opening (Figure 1G).

Finally, to address whether pocket accessibility is preserved in the tetramer, we conducted cryogenic electron microscopy (cryo-EM) experiments on the DC-SIGN ECD (Figure S5). Although resolution was low, the maps confirmed the expected oligomeric architecture with flexible CRD orientation, suggesting exposure of the secondary site under physiological conditions (Figure 1I–K and Figure S6).^32^ In a complementary experiment, ^1^H–^13^C transverse relaxation-optimized spectroscopy (TROSY) NMR experiments on ^13^C Met-labeled DC-SIGN ECD showed CSPs at M270 upon phenol addition, consistent with cryptic site accessibility in solution (Figure S7).

### Residues of the Secondary Site Are Hubs in a Ca^2+^ Responsive Allosteric Network

We have previously hypothesized that binding to the secondary site centered by M270 could allosterically modulate Ca^2+^-dependent carbohydrate binding of DC-SIGN.^28^ As a major hallmark of allosteric modulation is reciprocity, perturbations of the Ca^2+^-dependent CBS should propagate to the secondary site, detectable as CSPs in HSQC NMR spectra.^55^ Therefore, we recorded ^1^H–^13^C HSQC NMR spectra of ^13^C Met-labeled DC-SIGN CRD in the absence (apo) and presence (holo) of Ca^2+^, as well as under mannose-bound conditions. While mannose had no detectable effect, removal of Ca^2+^ induced CSPs and line broadening in the M270 side chain resonance, indicating conformational or dynamic changes that originate at the Ca^2+^ site and extend to the secondary site (Figure S8A,B). Notably, the CSP direction upon Ca^2+^ removal was opposite to that induced by phenol, and phenol addition to the apo protein shifted the signal back toward the holo state, resulting in a linear correlation of chemical shifts and supporting a model of conformational coupling between the Ca^2+^ sites and the cryptic pocket (Figure S8C).^56^ Complementing these observations, ^1^H–^15^N HSQC NMR titrations with CaCl_2_ revealed widespread CSPs and altered exchange regimes across the CRD, particularly in α2 and β2–4, underscoring global structural rearrangements (Figure S8D–F).

To gain deeper insight into how an allosteric signal could be communicated between the Ca^2+^ and the secondary site, we performed microsecond-scale all-atom MD simulations of both the apo and holo states of the CRD. We analyzed the normalized mutual information (NMI) between backbone dihedral angles (ϕ−ψ) and side chain dihedral angles (χ) for all pairs of residues, measuring the degree of conformational coupling, therefore revealing networks of communicating residues.^43,58^ While NMI graphs of the apo state revealed a more fragmented network, the holo state showed increased connectivity, especially within β2–4 and α2 (Figure 2A and Figure S9).

**Figure 2:**
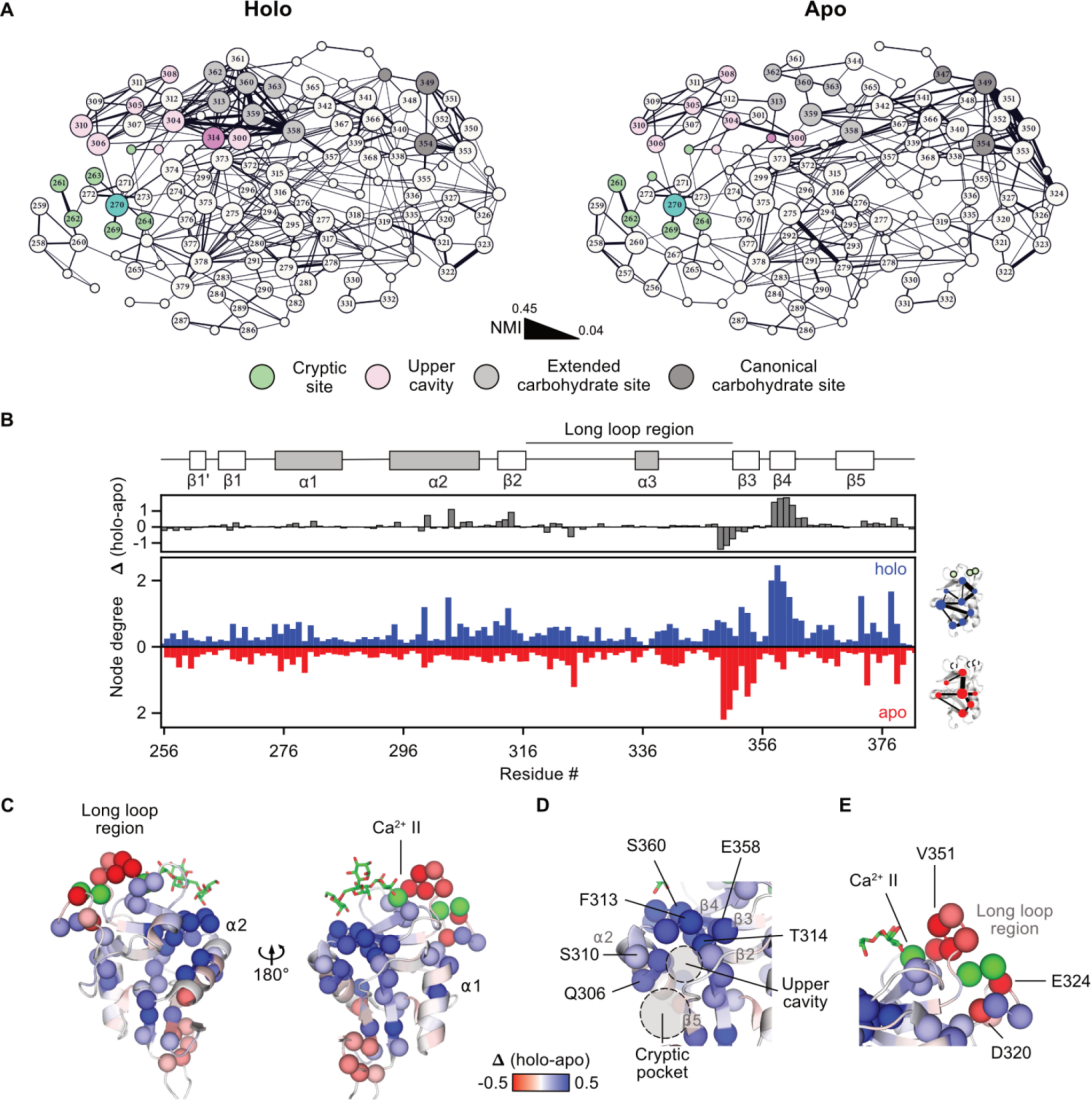
MD simulations and NMI analysis of apo and holo DC-SIGN reveal network hubs. (A) Network representation of the NMI graphs computed for the holo (left) and apo (right) states of DC-SIGN CRD. Edge thickness corresponds to computed NMI values (NMI threshold 0.04). Nodes exceeding a degree of 5 are defined as hubs and additionally as highlights if at least one edge exceeds an NMI value of 0.1. Nodes are scaled based on their importance in the network: Highlight and hub > highlight > hub > none. Nodes are colored according to their functional role in the CRD structure. Residues of the cryptic site and the upper cavity are shown in green and pink, respectively. Residues of the canonical and the extended CBS are shown in gray and light gray, respectively. A clear shift of connectivity toward the extended CBS is observed, when comparing the holo to the apo network (Figure S9). (B) Calculation of edge weighted node degrees from NMI analysis reveals residues with high importance in the holo (blue) and the apo (red) network of the CRD. The Δ node degree (holo – apo node degree) illustrates the importance of each residue in the holo network. (C) and (D) Mapping of Δ node degree on the CRD structure reveals clustering of holo network hubs at the extended carbohydrate site in β2–4 (E358, F359, S360, G361, N362, G363, and W364) and the upper cavity (T314, Q300, Q304, Q306, S307, and S310). (E) In contrast, negative Δ node degrees in residues of the long loop region indicate higher connectivity in the apo state.

This was further confirmed by comparing the node degrees as an indicator of the importance of each residue in the NMI networks.^58^ With the exception of a few long loop residues that showed higher node degree in the apo state, as previously reported for the CLR langerin, a broader set of residues in β2–4, α2, and the secondary site acted as network hubs in the holo state but drastically lost importance in the apo state (Figure 2B–E).^43^ Furthermore, analyzing hydrogen bond populations along the MD trajectories revealed significant changes in the same regions of the CRD, indicating that correlated conformations in the NMI network are underlying physical interactions (Table S1). Consistent with our observations from experimental CaCl_2_ titrations, these differences suggested Ca^2+^ binding enhanced both local and distant residue interactions. In particular, for residues lining the extended CBS and the upper cavity of the secondary site in β2–4 and α2, a high node degree coincided with conformational changes observed in our ^1^H–^15^N HSQC NMR CaCl_2_ titration experiments.

### Mutation of Hub Residues Alters Glycan Binding Properties of DC-SIGN

Overall, our data point to a Ca^2+^-responsive network that couples the Ca^2+^ sites to the cryptic pocket, with α2, the conserved hydrophobic core, and β3−β4 at the extended site as central nodes. Accordingly, we reasoned that perturbing NMI hubs or the cryptic pocket would disrupt the network, and tested eight CRD point mutants by ^1^H–^15^N HSQC NMR (Note S1).^55^ Among the selected mutants, the network hub residue T314A retained near-wildtype mannose affinity yet induced pronounced long-range CSPs > 10 Å away from the mutation site, when compared to the wildtype (Note S1 and Figures S11–S15). As T314 is located in the upper cavity where the M270 side chain rotates upon opening of the cryptic pocket, it lies central between other hubs of the NMI network located in α2, the conserved hydrophobic core, and β3 and β4 involved in the formation of the extended CBS (Figure 3A). This suggests that T314 also acts as a key residue in allosteric signal transmission from the secondary site. Although less prominent in the NMI analysis, we also selected the mutation M270F of the cryptic pocket gatekeeper residue M270 for experimental validation. As it mimics the cryptic pocket to be occupied by a small-molecule ligand similar to phenol, we reasoned that this could allow us to evaluate the effect of ligand binding on the allosteric network (Figure 3A).

**Figure 3:**
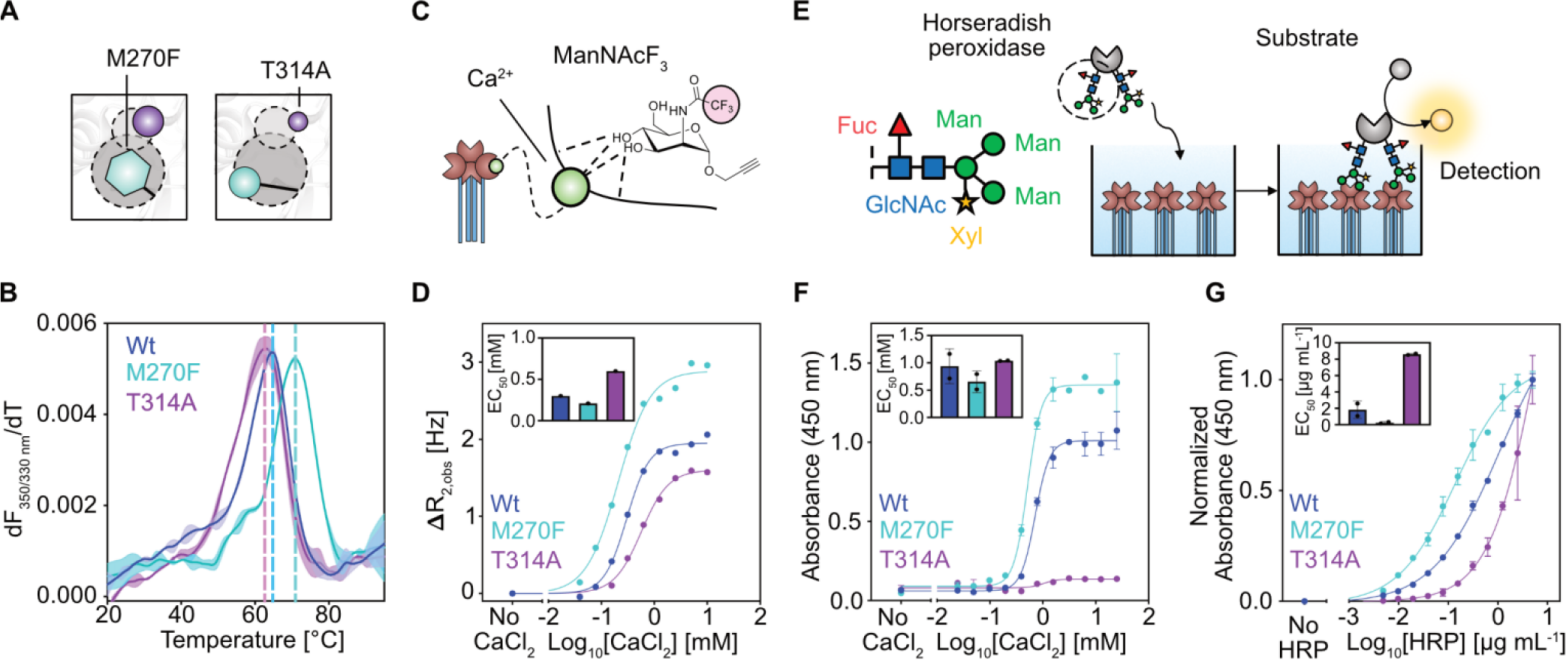
Mutations of network hubs T314 and M270 alter the glycan binding properties of DC-SIGN. (A) M270F mutant mimics pocket occupancy, while T314 in the upper cavity acts as a key holo network hub. (B) DSF curves of the ECDs revealed M270F (*T*_m_ = 70.4 ± 0.6 °C) to increase and T314A (*T*_m_ = 63.0 ± 0.4 °C) to slightly decrease thermal stability compared to the wildtype (*T*_m_ = 64.5 ± 0.1 °C). (C) Scheme of the ManNAcF_3_ reporter interacting with the canonical CBS. Ca^2+^-dependent binding of the reporter enables indirect measurement of Ca^2+^ affinity via R_2_-filtered ^19^F NMR.^28,57^ Adapted from ref 57. Copyright 2016, American Chemical Society. (D) Binding of the reporter under varying CaCl_2_ concentrations to the ECD proteins reveals minor differences in Ca^2+^ affinity but larger differences in maximum R_2,obs_. EC_50_ (hill slope): WT: 0.3 mM (2.0), M270F: 0.2 mM (1.4), T314A: 0.6 mM (1.7) (E) Multivalent binding of the oligosaccharide-carrying HRP enzyme to DC-SIGN ECD immobilized on a plate. Binding is evaluated using the peroxidase activity of HRP. Adapted from ref 31. Available under a CC-BY NC 4.0 license. Copyright 2025, Lefèbre et al. (F) Titration of CaCl_2_ at a constant HRP concentration to the ECD proteins confirms marginal changes in Ca^2+^ affinity. EC_50_ (mean ± SD): WT: 1.0 ± 0.4 mM, M270F: 0.7 ± 0.2 mM, and T314A: 1.2 ± 0.3 mM. (G) Titration of HRP at saturating CaCl_2_ concentration to the ECD proteins, reveal significant impact of the mutations on glycan binding abilities. EC_50_ (mean ± SD): WT: 1.8 ± 1.0 μg mL-1, M270F: 0.2 ± 0.2 μg mL^–1^, T314A: 8.6 ± 0.1 μg mL^–1^. Means and standard deviations of EC_50_ and *T*_m_ values for the HRP and the DSF assays were calculated from two biological replicates, each conducted in two technical replicates.

Considering their proposed importance in the Ca^2+^-dependent network, we hypothesized that the T314A and M270F mutations could affect folding, oligomerization, and Ca^2+^ affinity as well as the associated glycan binding characteristics of DC-SIGN. Successful purification of the ECDs via mannan affinity chromatography, dynamic light scattering (DLS), and differential scanning fluorimetry (DSF), indicated the mutants to be correctly folded and in their active tetrameric state, with M270F showing increased stability and T314A slightly reduced stability compared to wildtype (Figure 3B and Figure S16). To study the effect of the mutations on Ca^2+^-dependent carbohydrate binding activity, we measured binding of the fluorinated *N*-acetyl mannosamine analogue reporter (ManNAcF_3_) at a constant concentration under varying CaCl_2_ concentrations in ^19^F NMR R_2_-filtered NMR (Figure 3C).^28,57^ The obtained EC_50_ for the wildtype protein was in agreement with previously reported affinities for langerin, and a hill slope of >1 indicated cooperative effects as observed for other CLRs with accessory Ca^2+^ sites.^43,59,60^ While cooperativity was maintained in both mutants and apparent Ca^2+^ affinity shifted only marginally, the maximal R_2,obs_ at saturating CaCl_2_ was higher for M270F and lower for T314A than wildtype (Figure 3D). We orthogonally confirmed these observations based on CaCl_2_ titrations using the multivalent binding of the plate-immobilized ECDs to the glycoprotein horseradish peroxidase (HRP) (Figure 3E,F and Figure S17).^61^ Finally, titration of HRP at saturating CaCl_2_ concentrations revealed a 4-fold decreased affinity for T314A, while the M270F mutant showed 10-fold increased affinity compared to the wildtype (Figure 3G). Accordingly, while our binding assays suggested only marginal changes in Ca^2+^ affinity, perturbation of the allosteric network led to a marked shift in glycan binding, with T314A weakening and M270F strengthening engagement.

### The Allosteric Network Remodels the Extended Carbohydrate Binding Site

We observed only minor differences in mannose affinity between T314A, M270F, and the wildtype, and titration spectra showed nearly identical CSP trajectories for E347 and N349 of the EPN motif, indicating a conserved mannose binding mode at the canonical CBS (Figure 4A,C). Nevertheless, residues within the extended site on β3, β4, and α2, which were also identified as NMI hubs, exhibited pronounced CSPs and altered trajectories, especially in T314A (Figure 4B,C). The involvement of these NMI hubs supports allosteric coupling among the cryptic pocket, the upper cavity, and the extended site. Consistent with this view, changes in binding to glycosylated HRP could point toward remodeling of the extended CBS and altered oligosaccharide binding.

**Figure 4:**
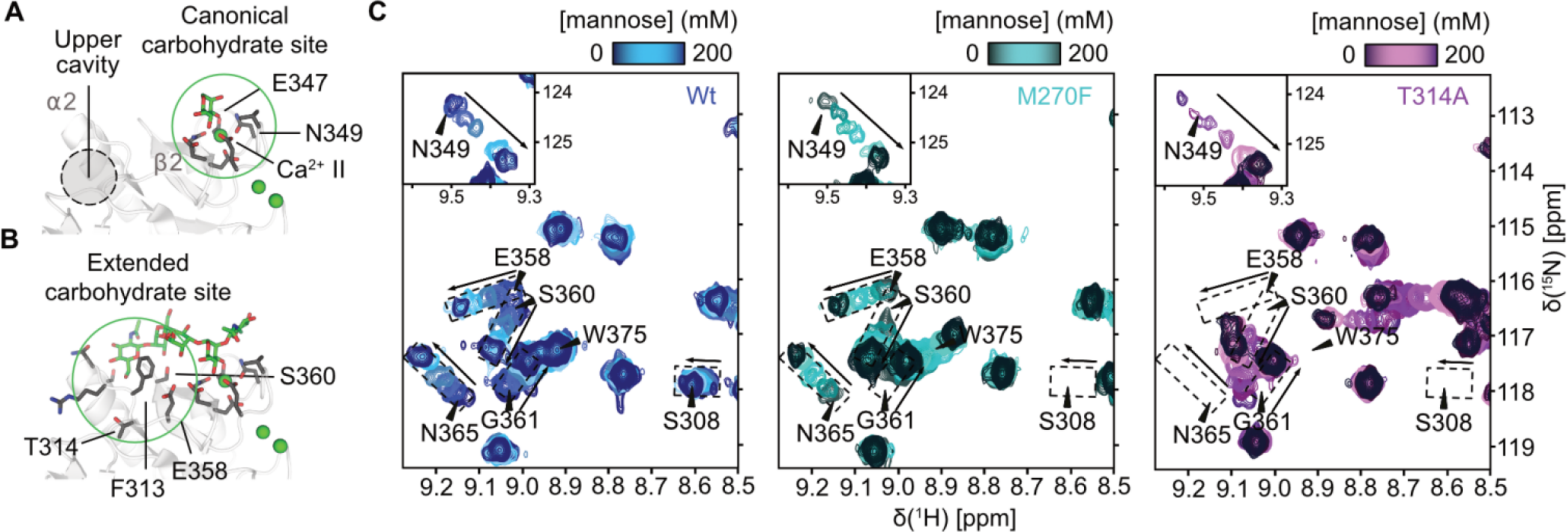
Mutations remodel the extended carbohydrate binding site. Interaction of mannose with the canonical CBS formed by Ca^2+^ site II and E347 and N349 of the EPN motif (A) and the GlcNAc_2_Man_3_ oligosaccharide with the extended CBS formed by β2–4, α2, and the α2−β2 connecting loop (B) (PDB ID: 1K9I). (C) ^1^H–^15^N HSQC mannose titrations show minor effects on the canonical CBS (e.g., residue N349) but larger CSPs and altered trajectories at the extended CBS (e.g., residues E358 and S360). Wildtype CSP trajectories are boxed and overlaid on mutant spectra.

Therefore, we tested binding of DC-SIGN to the Lewis X trisaccharide, previously shown to engage the extended CBS.^62^ ^1^H–^15^N HSQC NMR titrations revealed larger CSPs than those with mannose, with line broadening at residues such as F313 in the α2−β2 loop, consistent with stronger involvement of the extended CBS (Figures S18 and S19A,B). Although saturation was not reached, the estimated dissociation constant (*K*_D_) matched previously reported values (Figure S19C).^62^ M270F bound Lewis X more tightly, while T314A showed decreased affinity and altered CSP trajectories, indicating a different binding mode (Figures S19B,C and S20). Finally, ^1^H–^13^C HSQC NMR spectra of ^13^C Met-labeled wildtype under Lewis X-bound conditions revealed a CSP in the M270 resonance, supporting communication between the extended CBS and the cryptic pocket (Figure S21). Together, these results suggested that monosaccharide binding at the canonical Ca^2+^ site remains unchanged, whereas the cryptic pocket and upper cavity modulate oligosaccharide binding by reshaping the extended site, and that the conformations of both binding sites are reciprocally coupled.

### Network Hub Mutations Shift the Ca^2+^-Dependent Conformational Equilibrium of α-Helix 2

Our binding studies demonstrated skewing of the interaction of DC-SIGN with oligosaccharides toward lower or higher affinity by inserting mutations in the network. If this observation results from perturbation of the same allosteric network, their effects should be reflected in a concerted conformational response across structural elements involved in allosteric modulation.^63^

To test this, we superposed the ^1^H–^15^N HSQC NMR spectra of wildtype, M270F, and T314A and compared the direction and magnitude of chemical shift changes. Several resonances displayed linear displacement alongside a unifying chemical shift vector indicating exchange between two conformational states (Figure S22).^56^ The linear chemical shift correlation was most pronounced around the cryptic site in β1′ (e.g., T261, F262, F263, and Q264), in α2 (e.g., Q300 and F302), and in the α2−β2 connecting loop (e.g., R312) (Figure 5A). Structurally, an orbital π-stacking interaction between F263 and F302 anchors β1′ onto the rest of the CRD structure through α2, whose C-terminal end packs closely against β3 and 4 together with the α2−β2 loop. This provides a continuous surface for recognition of oligosaccharides at the extended CBS.^25^ While this could point to a mechanism in which oligosaccharide binding could be modulated by the relative positioning of α2 toward β3 and 4, several residues at the C-terminal end of α2 toward the upper cavity deviated from colinearity, indicating the presence of at least one additional state in that region (Figure 5A and Figure S22).^64^ Given that our MD simulations and the Ca^2+^ titration indicated that structural changes upon Ca^2+^ binding could potentially propagate to the upper cavity and the cryptic pocket via α2, we revisited this interaction and evaluated the relationship to our mutants by comparing the apo and holo ^1^H–^15^N HSQC NMR spectra of all three proteins (Figure 5B). Intriguingly, we observed continuation of the linear shift trajectories in several residues, such as F262, F263, Q264, and F302, demonstrating a concerted structural response to the M270F and T314A mutations and Ca^2+^ binding. In line with our MD simulations, this suggested that a Ca^2+^-responsive network is at play that can be perturbed by mutating the hub residues.

**Figure 5:**
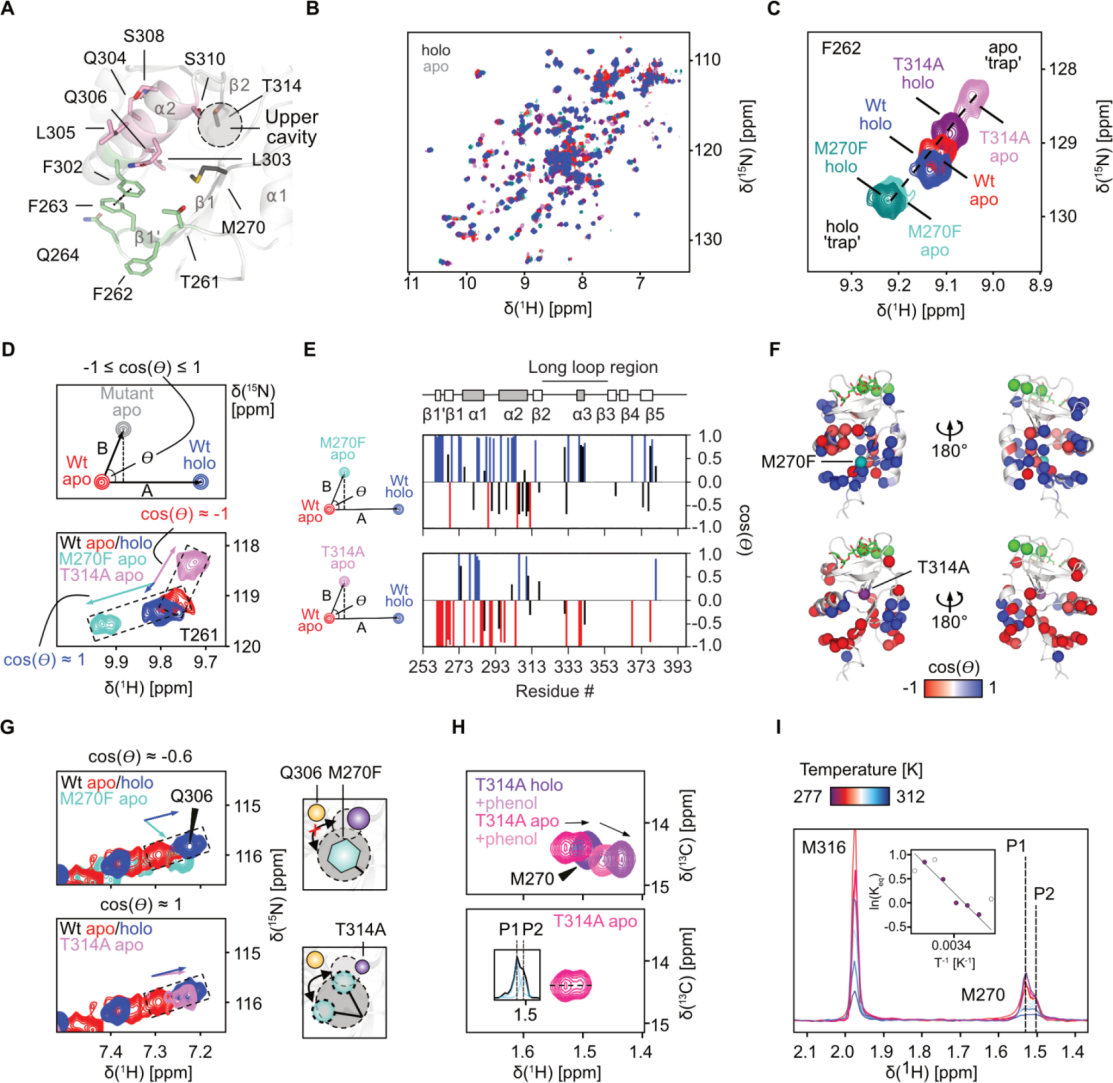
Mutations shift the conformational equilibrium of DC-SIGN by altering the open-closed equilibrium of the cryptic pocket. (A) Residues showing colinear displacement in ^1^H–^15^N HSQC NMR spectra upon T314A or M270F mutation (green), versus nonlinear residues at the α2 C-terminal end/upper cavity (red). F263 and F302 form a π-stacking interaction, anchoring β1′ to α2 and the rest of the CRD (Figure S22). (B) Overlay of apo and holo ^1^H–^15^N HSQC NMR spectra of the wildtype and the mutant CRDs. (C) Example β1′ resonance following colinear CSPs. T314A shifts beyond the apo state (apo "trap"), while M270F shifts beyond the holo state (holo “trap”). (D) Vector scheme of CHESPA to determine the angle θ between chemical shift vectors of apo to holo wildtype and apo wildtype to apo mutant. A cos­(θ) ≈ 1 indicates conformational similarity to holo wildtype, while cos­(θ) ≈ −1 suggests similarity to the apo wildtype conformation.^65^ The resonance of T261 is shown as an example. (E) and (F) CHESPA analysis of the apo and holo spectra revealed resonances of residues in β1′, α2, and the cryptic site to approach cos­(θ) ≈ 1 for M270F but showed cos­(θ) ≈ −1 for T314A, indicating a shift toward the holo and apo state, respectively. Yet, residues of the upper cavity deviate from this trend. (G) This is exemplified by the Q306 chemical shift (left) showing T314A to induce a holo-like state (cos­(θ) ≈ 1) in this residue, while the Q306 resonance in M270F showed nonlinear arrangement (cos­(θ) ≈ −0.6). The scheme shows the proposed constitutive closing or exposure of the pocket for M270F or T314A, respectively. (H) ^1^H–^13^C HSQC NMR spectra of apo and holo ^13^C Met-labeled DC-SIGN CRD T314A interacting with phenol (top) do not show a unifying shift vector as observed for the wildtype (Figure S8C). The apo spectrum (bottom) shows a split resonance for M270, indicating two slowly interchanging states, P1 and P2, that resemble the apo and holo state, respectively. Extraction of the ^1^H dimension of the apo spectrum (inset) allows for line fitting of the populations as shown in (I). (I) Line fitting apo spectra at increasing temperatures shows a shift toward P1. Van’t Hoff analysis (inset) indicates an endothermic transition (ΔH = +32 kJ mol^–1^) and a positive entropy change (ΔS = +112 J mol^–1^ K^–1^). Uncolored data points were not used for fitting. ^1^H–^13^C HSQC NMR spectra of the resonance at different temperatures are shown in Figure S24.

Along trajectories of resonances of residues at the interface of β1′, α2, and the cryptic site, the apo M270F resonances shifted beyond the respective holo wildtype resonances, suggesting the mutant to stabilize a holo-like state, while the T314A mutation appeared to stabilize an apo-like state. As neither M270F nor T314A recovered the wildtype holo state upon removal or addition of Ca^2+^ in these resonances, respectively, we speculated that the effect induced by the mutations could trap α2 in either a holo or an apo-like conformation independent of Ca^2+^ (Figure 5C). Taken together, this indicated that the cryptic pocket and upper cavity skew the Ca^2+^ responsive two-state equilibrium of α2, thereby modulating the extended site while leaving the canonical CBS and its Ca^2+^ site largely unchanged.

### The T314A Mutation Shifts the Open-Closed Equilibrium of the Cryptic Site toward the Open State

To evaluate the Ca^2+^-dependent two-state equilibrium of the CRD systematically, we compared the conformational similarity of holo and apo wildtype and the apo mutant spectra using chemical shift projection analysis (CHESPA) of the ^1^H–^15^N HSQC NMR spectra. CHESPA compares CSP vectors from mutations or other perturbations to those from an orthosteric reference and uses the cosine of the vector angle θ to quantify conformational similarity to the active or inactive state of the protein (Figure 5D).^65^ Overall, this revealed M270F and T314A to shift the CRD toward the holo state and apo state, respectively (Figure 5E,F). However, this effect did not apply to the C-terminal end of α2 and the upper cavity, as T314A induced a holo-like state in Q306, while in M270F, the chemical shift changes deviated from colinearity (Figure 5G). As a previous X-ray structure of DC-SIGN in the open state predicted a hydrogen bond of Q306 to the M270 thioether side chain, we hypothesized that this observation could reflect the inability of the M270F mutant to open the cryptic pocket by rotation of residue 270 into the upper cavity (Figure 5G and Figure S23).^66^ Contrastingly, as we have observed the M270 side chain to resemble the holo state upon phenol binding to apo DC-SIGN wildtype in our ^1^H–^13^C HSQC NMR experiments, we reasoned that the holo-like state of Q306 in T314A could indicate a shift in the open-closed equilibrium toward the open species, therefore exposing the hydrophobic cryptic pocket even in the absence of a ligand (Figure 5G).

As the open-closed conformational equilibrium, and with it the chemical shift of Q306, should be coupled to the M270 side chain dynamics, we recorded ^1^H–^13^C HSQC NMR spectra of apo and holo ^13^C Met-labeled T314A in the presence and absence of phenol and compared them to experiments with the wildtype. In line with our hypothesis of a preferred open state upon mutation, we observed a shift of the M270 resonance in holo T314A beyond the resonance of the wildtype bound to phenol. In the presence of phenol, the T314A trajectory is continued with a CSP of higher magnitude, suggesting that T314A preserves the ability to interact with ligands at the cryptic site (Figure 5H, top). Intriguingly, removing Ca^2+^ from T314A revealed a split in the M270 resonance, with a higher populated state that resembled the wildtype bound to phenol and a second state overlapping with the holo T314A M270 resonance (Figure 5H, bottom). This aligns with our CHESPA data, indicating that even without Ca^2+^, M270 can adopt a holo-like conformation, with the two distinct peaks in apo spectra indicating slow interconversion between these conformations on the NMR time scale. Analysis of the temperature dependency of the chemical shift showed a trend toward the apo state above ∼298 K, consistent with an entropically driven, endothermic transition confirmed by Van’t Hoff analysis, with Ca^2+^ release increasing the conformational freedom of the cryptic pocket (Figure 5I and Figure S24). Together with the CHESPA results and the slow-exchange behavior, the thermodynamic profile strongly suggested that the Ca^2+^-dependent conformational state of M270 in apo T314A is already biased toward an open conformation, partially decoupling the phenol bound from the Ca^2+^ bound conformation. This was further supported by the nonlinearity of the CSPs when comparing spectra of apo and holo T314A with and without phenol, indicating that phenol binding perturbs an already open conformation (Figure 5H and Figure S8C).

Collectively, our NMR data strongly suggested that the mutants T314A and M270F favor an open state or mimic an occupied state, respectively. Thus, we reasoned that the concerted chemical shift response to Ca^2+^ and the mutations could directly report on the populations of the exposed and bound state of the cryptic pocket.^67^ This would indicate that binding to the pocket could shift the structure to a rather Ca^2+^ bound state, which would, per our NMI analysis and affinity measurements, lead to a higher connectivity and affinity of the extended CBS. In turn, exposure of the hydrophobic pocket to the solvent, as seen in T314A, would destabilize the network at the extended CBS, leading to decreased affinities. Retrospective analysis of published DC-SIGN CRD X-ray structures supported this model, with pocket occupation associated with increased stability around α2 and β3–4 (Note S2 and Figure S25). Finally, in line with a stabilizing effect of closing the cryptic pocket, we found that phenylalanine seems to be evolutionarily favored across human CLRs at the position equivalent to M270 in DC-SIGN (Figure S26).

## Discussion

DC-SIGN-glycan interactions are critical for pathogen recognition, immune modulation, and cellular adhesion.^4,5,36,38,68,69^ Uncovering how these interactions are regulated at a molecular level offers critical insight into how glycan recognition by the DC-SIGN CRD translates into downstream cellular response, informing DC-SIGN-targeted drug discovery. Past work has identified a secondary site that was proposed to allosterically activate carbohydrate binding in DC-SIGN through interactions with a bivalent glycomimetic ligand, but the underlying mechanism remained elusive.^28^ Here, we demonstrate that binding to this site underlies cryptic pocket opening, allosterically activating glycan binding via the extended CBS of DC-SIGN.

We propose a model wherein the occupation state of the cryptic pocket modulates glycan binding affinity through conformational changes in α2, adopting either a holo-like or an apo-like state depending on pocket occupancy. Central to this mechanism is M270, a gatekeeper residue whose side chain rotation into an upper cavity enables cryptic pocket opening, exposing a hydrophobic cleft. We identified the M270F and T314A mutations as proxies for the occupied and unoccupied states of the cryptic pocket, increasing or decreasing the glycan affinity, respectively. These phenotypes are linked to a concerted structural response, skewing the Ca^2+^-dependent conformational equilibrium of DC-SIGN, specifically affecting α2. While T314A traps this region in an apo-like state, M270F stabilizes a conformation resembling the holo state of the wildtype, even in the absence of Ca^2+^. The helix therefore behaves as a two-state hinge coupling pocket occupancy to glycan recognition.

While interactions with the Ca^2+^-coordinated EPN motif remain largely unchanged in the mutants, our NMR studies, MD simulations, and glycan binding assays point to the extended CBS as the major site of allosteric regulation. Residues at the interface of α2 and β2–4, including F313, S360, and E358, showed pronounced CSPs upon mutation and were identified as high-connectivity nodes in the network. Previous structural studies have shown that the same residues contribute to glycan specificity and affinity by forming secondary contacts that enable DC-SIGN to accommodate oligosaccharides in distinct geometries.^25,27^ In contrast, interactions of monosaccharides at the canonical CBS are less variable, and, independent of the glycan involved, uniformly interact with the same hydrogen bond acceptors and donors via their 3-OH and 4-OH groups.^70^ These observations suggest that the allosteric mechanism we describe modulates glycan affinity and selectivity by priming and potentially reshaping the extended CBS.

We found that Ca^2+^ complexation assumed a structural role beyond direct interactions with monosaccharides. Both our MD simulations and NMR studies showed apo DC-SIGN to sample a broader conformational ensemble, similar to what has been described for the related CLRs DC-SIGNR and langerin.^43,71^ Binding to Ca^2+^ leads to a reduced rate of interconversion between conformations and higher connectivity, especially affecting residues of the extended site and α2, and also the open-closed conformation of the cryptic pocket. Accordingly, cofactor complexation potentially has a similar effect on DC-SIGN as occupation of the cryptic site, that is, priming the extended CBS for interactions with oligosaccharides. We hypothesize that this effect is mediated by a network of interactions, including an orbital π-π interaction between F263 and F302. Similar π-stacking interactions have been implicated in the oligomerization behavior of the related CLR langerin.^72^ Yet, DC-SIGN and langerin oligomers display distinct topology and relative orientation of CRDs, and we observed both mutants to form tetramers similar to the wildtype.^33,73^ While it is possible that this interaction impacts the relative spatial arrangement of CRDs without affecting tetramerization via the neck domain, changes in affinity of both ECD and CRD suggested intradomain allosteric control.^32^ Supporting this mechanism, pocket occupation results in reduced flexibility in α2 and β3 and 4 and increased stability, as suggested by melting temperatures and evolutionary preference for a phenylalanine in the position of M270. While reduced flexibility of the CBS as affinity-driving factor in CLRs has not been investigated so far, studies on other glycan binding proteins, for instance galectins, have shown that a preorganized carbohydrate recognition domain and conformationally restricted glycan ligands can substantially diminish the entropic penalty associated with binding of conformationally flexible glycans, thereby enhancing overall affinity and shaping selectivity.^74,75^

While the dominant two-state equilibrium is centered around α2, several observations also pointed toward extensive dynamics connecting the canonical CBS, the extended site, and the Ca^2+^ sites. First, residues such as E358, S360, and F313 show large mutation-induced CSPs that did not follow linear chemical shift behavior, suggesting the presence of intermediate states beyond a simple two-state model.^64^ Second, these same residues are also perturbed upon monosaccharide binding, despite being spatially distant from the canonical Ca^2+^-coordinated site, indicating that the canonical and extended sites are also structurally linked.^12^ Third, previous studies on the closely related CLR DC-SIGNR have shown interdependence of Ca^2+^ binding at the accessory Ca^2+^ sites and glycan binding.^71,76^ Therefore, subtle changes in the Ca^2+^ affinity of DC-SIGN in our binding assays might also be rooted in our setup, only indirectly measuring Ca^2+^ affinity via glycan binding. Finally, as we observed positive cooperativity between the Ca^2+^ sites of the CRD, several additional conformations of the long loop region and adjacent sites are possible.^59,60^

Taken together, our observations draw a complex picture of hierarchical allosteric modulation of DC-SIGN with both local and global dynamics differentially affecting the outcome of glycan binding. Previous studies on DC-SIGN have described a relationship between glycan binding dependent effects on both endocytosis and signaling.^3,36,38,39,77^ Thus, it is tempting to speculate whether the allosteric mechanism described here also translates into broader receptor-level response under physiological conditions as suggested for other CLRs.^40,41^

Although the currently available data did not allow us to clearly separate the different effects and their contributions, the integration of an allosteric cryptic site into the network suggests that this complex interplay can be modulated by small molecules. In this context, our results also highlight the potential of combining data from simulations with subsequent experimental validation by NMR to identify hidden cryptic sites in proteins with low druggability. Cryptic pockets have emerged as useful entry points for ligand discovery targeting proteins with primary sites of low druggability, and evidence from other CLRs suggests that structurally analogous pockets may occur more broadly within the C-type lectin-like domain (CTLD) fold.^46,78^ In support of this, our sequence analysis showed conservation of several residues that shape this pocket, including the position corresponding to M270 and the surrounding framework residues, suggesting that the structural capacity to form such a cavity is maintained across multiple CLRs. At the same time, the availability of the pocket will depend on whether the upper cavity, showing much lower sequence conservation, can accommodate the rotating side chain, as seen in DC-SIGN. For example, a structurally equivalent allosteric cryptic pocket has been described in the Ca^2+^-independent CLR NKG2D, where the same cavity forms through rotation of F113 in a manner that mirrors the movement of M270 in DC-SIGN.^79^ These observations indicate that the structural capacity for such a cryptic site may be conserved, although its functional accessibility is likely CLR-specific and requires further comparative analysis.

Taken together, while secondary sites and in some cases allosteric mechanisms have been described for other CLRs, the present study is, to the best of our knowledge, the first description of a cryptic pocket and its functional effect identified in a glycan binding CLR.^28,46,47,49,51,80^ Building on previously identified fragment hits that engage this region, the cryptic pocket conformation defined here now provides a structural basis for future efforts aimed at designing small-molecule allosteric modulators of DC-SIGN. By enabling selective tuning of extended CBS engagement without disrupting Ca^2+^ coordination or glycan specificity at the canonical CBS, targeting the site identified here could, for example, enable fine-tuning of cell–cell and cell-pathogen interactions, eventually tuning the immunological response to specific glycans. As other glycan binding CLRs suffer from low druggability similar to DC-SIGN, we envision our approach to equally stimulate drug discovery campaigns targeting other pharmaceutically relevant members of this protein family.^45^

## Supplementary Material



## Notes

Experimental procedures, supplementary notes describing mutant selections (Note S1) and retrospective analysis of X-ray structures (Note S2), supplementary Figures S1–S26, and supplementary Table S1 (PDF)

## †

Recursion, 41S Rio Grande Street, Salt Lake City, UT 84101, USA (S.A.)

## Abbreviations

DC-SIGN: dendritic cell-specific intercellular adhesion molecule-3-grabbing nonintegrin
CLR: C-type lectin receptor
CRD: carbohydrate recognition domain
CBS: carbohydrate binding site
ECD: extracellular domain
MD: molecular dynamics
NMR: nuclear magnetic resonance
cryo-EM: cryogenic electron microscopy
DLS: dynamic light scattering
DSF: differential scanning fluorimetry
CHESPA: chemical shift projection analysis
NMI: normalized mutual information
