# Supplementary material for: A Cryptic Pocket Allosterically Modulates Oligosaccharide Binding to DC-SIGN
Source: JACS Au. 2026 Jan 20;6(2):1048–60. doi: 10.1021/jacsau.5c01465 (PMC12933331; doi:10.1021/jacsau.5c01465)
Supplement: Supplementary file 1 [file au5c01465_si_001.pdf]

SUPPORTING INFORMATION

A Cryptic Pocket Allosterically Modulates Oligosaccharide Binding to DC-SIGN

Jonathan Lefèbre[1, 2, 3], Maurice Besch[1, 2, 3], Marcelo Daniel Gamarra[4, 5], Jan-Oliver Kapp-Joswig[6], Annika Balke[7], Stevan Aleksić[6, †], Henry Flatau[1, 3], Gregor Suchy[1, 3], Elena Georgieva[6], Patrick Scheerer[7], Bettina G. Keller[6], Carlos Pablo Modenutti[4, 5, 8], Christoph Rademacher[1, 3, *]

[1] Department of Pharmaceutical Sciences, University of Vienna, Josef-Holaubek-Platz 2, Vienna 1090, Austria,

[2] Vienna Doctoral School of Pharmaceutical, Nutritional and Sport Sciences, University of Vienna, Josef-Holaubek-Platz 2, Vienna 1090, Austria,

[3] Department of Microbiology, Immunology and Genetics, University of Vienna, Max F. Perutz Labs, Dr. Bohr Gasse 9, Vienna 1030, Austria,

[4] Departamento de Química Biológica, Facultad de Ciencias Exactas y Naturales, Universidad de Buenos Aires (FCEyN-UBA), Ciudad de Buenos Aires C1428EGA, Argentina,

[5] Instituto de Química Biológica de la Facultad de Ciencias Exactas y Naturales (IQUIBICEN) CONICET, Pabellón 2 de Ciudad Universitaria, Ciudad de Buenos Aires C1428EHA, Argentina,

[6] Freie Universität Berlin, Department of Biology, Chemistry, Pharmacy, Arnimallee 22, Berlin 14195, Germany,

[7] Charité - Universitätsmedizin Berlin, corporate member of Freie Universität Berlin, Humboldt-Universität zu Berlin, Institute of Medical Physics and Biophysics, Group Structural Biology of Cellular Signaling, Berlin 10117, Germany,

[8] Istituto di Biologia e Biotecnologia Agraria (IBBA), Consiglio Nazionale della Ricerca (CNR), Via Alfonso Corti nr. 12, Milano 20133, Italia

***Corresponding author: Christoph.Rademacher@univie.ac.at**

[†]**Present Addresses:** Recursion, 41S Rio Grande Street, Salt Lake City, UT 84101, USA

Table of contents

Experimental procedures 2

Supplementary notes 11

Supplementary figures 13

Supplementary tables 32

Supplementary references 33

Experimental procedures

Protein Expression and Purification

General remarks. Unless stated otherwise, all chemicals, growth media and enzymes used for protein expression and purification were purchased from Sigma Aldrich or Carl Roth. Codon-optimized genes for the bacterial expression of wildtype and mutant DC-SIGN CRD and ECD were purchased from GenScript.

DC-SIGN carbohydrate recognition domain. ^{15}N-labeled DC-SIGN CRD wildtype and mutants were produced as previously described.[1] In brief, the protein was expressed insolubly in BL21 (DE3) E. coli transformed with a pET28a vector encoding amino acids 253-404 of DC-SIGN and a N-terminal His-tag. Bacteria grown in M9 minimal medium supplemented with 35 mg L^{-1} kanamycin and 0.5 g L^{-1} $^{15}NH_4Cl$ were induced at OD_{600} of 0.9 using IPTG, lysed and inclusion bodies were harvested by centrifugation. Following solubilization, the protein was refolded overnight via rapid dilution. Next, the protein was dialyzed against 50 mM Tris-HCl, 150 mM NaCl, 10 mM $CaCl_2$ (pH 7.8) and purified via Ni^{2+} NTA affinity chromatography. Purified protein was pooled and dialyzed against 20 mM MES, 40 mM NaCl, 10 mM $CaCl_2$ (pH 6.0), concentrated using a centrifugal spin filter, snap frozen and then stored at -80°C.

^{13}C Met-labeled DC-SIGN CRD wildtype, M270F and T314A were produced following the same protocol as described for ^{15}N-labeled DC-SIGN CRD, with the exception that 0.1 g L^{-1} ^{13}C ε-methyl methionine was added to the M9 minimal medium at OD_{600} of 0.45 to avoid scrambling of the isotope.

DC-SIGN extracellular domain. DC-SIGN ECD wildtype, M270F and T314A were produced as previously described.[1] In brief, the protein was expressed insolubly in BL21 (DE3) E. coli transformed with a pET30b vector encoding amino acids 64-404 of DC-SIGN. Bacteria were grown in Luria-Bertani (LB) medium with 35 mg L^{-1} kanamycin and expression was induced with IPTG at OD_{600} of 0.9. Following lysis, inclusion bodies were harvested by centrifugation and solubilized. The protein was refolded via rapid dilution and dialyzed against 25 mM Tris-HCl, 150 mM NaCl, 25 mM $CaCl_2$, pH 7.8, for subsequent purification via mannan agarose affinity

chromatography. Purified protein was dialyzed against 25 mM HEPES, 150 mM NaCl, 10 mM $CaCl_2$, pH 7.4, concentrated using a centrifugal spin filter, snap frozen and then stored at -80°C.

^{13}C ε-methyl methionine-labeled DC-SIGN ECD was expressed following the same protocol as described for DC-SIGN CRD by adding 0.1 g L^{-1} ^{13}C ε-methyl methionine to the M9 minimal medium at OD_{600} of 0.45. Following expression, the protein was purified as described for the unlabeled ECD. The tetrameric state of the purified proteins was confirmed using DLS measurements at 0.1 mg mL^{-1} protein concentration on a Zetasizer Advance Pro (Malvern Panalytical). Melting temperatures T_m were measured at 0.1 mg mL^{-1} protein concentration in thermal shift assays on a NanoTemper Prometheus (NanoTemper).

Plate-based horseradish peroxidase assay

General remarks. Unless stated otherwise, all reagents and buffers were obtained from Sigma Aldrich or Carl Roth. Measurements were done on a Enspire Multimode Plate Reader (PerkinElmer). GraphPad Prism was used for all data processing and analysis.

$CaCl_2$ titration. 100 µg mL^{-1} DC-SIGN ECD were immobilized in immobilization buffer (25 mM HEPES, 150 mM NaCl, 25 mM $CaCl_2$, pH 7.4) overnight at 4°C on transparent Nunc Maxisorp 96-well plates (Thermo Fisher). Protein solution was removed, and plates were washed three times with immobilization buffer and then blocked for 2 hours at 4°C with blocking buffer (25 mM HEPES, 150 mM NaCl, 25 mM $CaCl_2$, 2% BSA, 0.01% Tween-20). Plates were washed twice with 25 mM HEPES, 150 mM NaCl, 5 mM EDTA, pH 7.4 and twice with 25 mM HEPES, 150 mM NaCl, pH 7.4 to remove residual Ca^{2+}. To titrate Ca^{2+}, $CaCl_2$ in 25 mM HEPES, 150 mM NaCl, pH 7.4 was serially diluted on the plate at a constant HRP concentration of 1 µg mL^{-1} and then incubated at RT for 2 hours. Following incubation, plates were washed three times with 25 mM HEPES, 150 mM NaCl, pH 7.4 and bound HRP was detected using a TMB substrate kit (Thermo Fisher) and 0.18 M H_2SO_4 according to manufacturer's instructions. Absorption was measured at 450 nm.

HRP titration. Immobilization and blocking were done as described above. After blocking, plates were washed three times with immobilization buffer. HRP in immobilization buffer was serially diluted on the plate at a constant concentration $CaCl_2$ concentration of 25 mM and then incubated

at RT for 2 hours. Following incubation, plates were washed three times with immobilization buffer and HRP was detected as described above.

Mannose titration. Immobilization and blocking were done as described above. After blocking, plates were washed three times with immobilization buffer. Mannose in immobilization buffer containing 1 µg mL^{-1} HRP was serially diluted on the plate and then incubated at RT for 2 hours. Following incubation, plates were washed three times with immobilization buffer and HRP was detected as described above.

All conditions were measured in technical triplicates, averaged and normalized to the control well containing only buffer without ligand ($CaCl_2$, HRP or mannose). IC_{50} and EC_{50} values and hill slopes (n_H) were obtained in a four-parameter fit according to equation (**1**).

$$Y = \text{bottom} + \frac{\text{top} - \text{bottom}}{1 + 10^{n_H(\text{LogEC}_{50} - X)}} \qquad (1)$$

With bottom and top representing the minimal and maximal response plateaus, respectively, Y as the observed response and X the logarithmic concentration of the ligand.

NMR spectroscopy

General remarks. ^1H-^{13}C HSQC NMR, ^1H-^{15}N HSQC NMR, ^1H-^{15}N SOFAST-HMQC NMR and ^{19}F NMR measurements were conducted on an Ultra Shield 500 MHz spectrometer (Bruker) equipped with a TCI H/F-C-N Prodigy probe. If not state otherwise all ^1H-^{13}C HSQC NMR, ^1H-^{15}N HSQC NMR and ^1H-^{15}N SOFAST-HMQC NMR spectra were collected at 298 K. ^1H-^{13}C TROSY NMR measurements were conducted on an Ascend 700 MHz spectrometer (Bruker) equipped with a TCI H/F-C-N Helium CryoProbe at 298 K. All samples were measured at sample volumes of 160 µL in 3 mm NMR tubes (Bruker). Unless stated otherwise, all reagents and buffers were obtained from Sigma Aldrich or Carl Roth. Lewis X trisaccharide was obtained from Biosynth. Spectra were processed in TopSpin 4.2.0 and data analysis was performed using CCPN Analysis 3.2.0 for 2D spectra and in MestreNova for 1D spectra.[2] Further analysis was done using GraphPad Prism and in-house python scripts.

^{1}H-^{13}C HSQC NMR. ^{1}H-^{13}C HSQC NMR spectra were collected with 512 increments in the carbon and 8 scans per increment and 2048 points in the direct dimension. The relaxation delay d1 was set to 1.5 s. The W5 WATERGATE pulse sequence was used for solvent suppression[3]. Experiments were performed at a CRD concentration of 200 µM in 20 mM MES, 40 mM NaCl, 10 mM $CaCl_2$ (pH 6.0) supplemented with 10% D_2O and, if applicable, 20 mM phenol, 20 mM mannose or 10 mM Lewis X. For spectra of apo DC-SIGN CRD and $CaCl_2$ titrations, Ca^{2+} was removed as previously described by dialyzing the protein against 20 mM MES, 40 mM NaCl, 1 mM EDTA (pH 6.0) twice, and then against 20 mM MES, 40 mM NaCl (pH 6.0) twice prior to sample preparation and measurements[1]. Reference spectra containing only buffer were used to evaluate potential scrambling of the ^{13}C ε-methyl methionine (Figure S3).

The temperature-dependent population shift of the M270 resonance was analyzed by extracting populations P1 and P2 by applying a general Lorentzian line fitting to the extracted ^{1}H projections of ^{1}H-^{13}C HSQC NMR spectra recorded at different temperatures. Thermodynamic parameters were obtained from fitting a Van't Hoff plot according to equation 2.

$$\ln K_{eq} = -\frac{\Delta H}{R} \cdot \frac{1}{T} + \frac{\Delta S}{R} \tag{2}$$

With $\ln K_{eq}$ as the natural logarithm of the equilibrium constant of the populations (P1/P2), **Δ**H the enthalpy change (in J mol^{-1}), **Δ**S the entropy change (in J mol^{-1} K^{-1}), R the gas constant (8.314 J mol^{-1} K^{-1}) and T the absolute temperature in K.

^{1}H-^{13}C TROSY NMR. ^{1}H-^{13}C TROSY NMR spectra were acquired at a sample temperature of 298 K with 128 increments in the carbon and 16 scans per increment and 2048 points in the direct dimension. The relaxation delay d1 was set to 0.45 s. The W5 WATERGATE pulse sequence was used for solvent suppression [3]. Experiments were performed at a ECD concentration of 300 µM in 25 mM HEPES, 150 mM NaCl, 10 mM $CaCl_2$ (pH 7.4) supplemented with 10% D_2O and, if applicable, 20 mM phenol.

^{1}H-^{15}N HSQC NMR. ^{1}H-^{15}N HSQC NMR spectra were collected with 256 increments in the nitrogen and 24 scans per increment and 2048 points in the direct dimension. The relaxation delay d1 was set to 1.0 s. The W5 WATERGATE pulse sequence was used for solvent suppression.[3, 4] Experiments were performed at a CRD concentration of 200 µM in 20 mM MES, 40 mM NaCl, 10 mM $CaCl_2$ (pH 6.0) supplemented with 10% D_2O and, if applicable, varying concentrations of ligands. Samples of apo DC-SIGN CRD were prepared as described for ^{1}H-^{13}C HSQC NMR experiments. A previously published resonance assignment of DC-SIGN CRD was transferred to the nearest neighbor in a reference spectrum recorded without ligand.[5] Unassigned, overlapping or disappearing peaks were not assigned.

CSPs induced by mutations or ligands were calculated as previously described according to equation 3.[6]

$$\mathrm{CSP} = \sqrt{\frac{\left(\delta(^{1}\mathrm{H})\right)^2 + \left(\alpha \cdot \delta(^{15}\mathrm{N})\right)^2}{2}} \tag{3}$$

The empirical weighing factor a was set to 0.15 for all calculations.

Dissociation constants (K_Ds) were determined in titration experiments at five ligand concentrations $[L]_T$ for mannose and at three for Lewis X at constant a protein concentration $[P]_T$. Only residues in the fast exchange regime were used for the fitting procedure via equation 4 in a global two-parameter fit.

$$\mathrm{CSP} = \mathrm{CSP}_{\mathrm{max}}\, p_B \tag{4}$$

with CSP_{max} corresponding to the CSP values upon saturation and the bound protein fraction p_b corresponding to

$$p_B = \frac{[P]_T + [L]_T + K_D - \sqrt{([P]_T + [L]_T + K_D)^2 - 4[P]_T[L]_T}}{2[P]_T}$$

CHESPA was performed as previously described.[7] The chemical shift vector between apo and holo wildtype DC-SIGN CRD was used as reference perturbation to calculate the vector product to

determine cos(θ) of the vector between apo mutant and apo wildtype. Only residues showing CSP > 0.015 ppm were used for calculation.

^{1}H-^{15}N SOFAST-HMQC NMR. ^{1}H-^{15}N SOFAST-HMQC NMR spectra were collected with 200 increments in the nitrogen and 32 scans per increment and 1024 points in the direct dimension. The relaxation delay d1 was set to 0.3 s.[8] Sample preparation and CSP analysis was done as described for the ^{1}H-^{15}N HSQC NMR experiments.

^{19}F R_2-filtered NMR reporter displacement assay. The ^{19}F R_2-filtered NMR reporter displacement assay was adjusted for $CaCl_2$ titrations with DC-SIGN ECD as previously described.[1] Apparent transverse relaxation rates $R_{2,obs}$ were obtained using the CPMG pulse sequence with a relaxation delay d1 of 2.0 s, acquisition time t_{acq} of 0.8 s and a CPMG frequency ν_{CPMG} of 500 Hz. DC-SIGN ECD was dialyzed four times against 25 mM HEPES, 150 mM NaCl (pH 7.4) to remove Ca^{2+} prior to measurements. Samples contained 25 µM DC-SIGN ECD in 25 mM HEPES, 150 mM NaCl (pH 7.4) supplemented with 10% D_2O and 0.1 mM of the $ManNAcF_3$ reporter molecule. TFA at a concentration of 0.1 mM served as an internal reference. 512 scans were recorded to ensure sufficient signal to noise ratios. $R_{2,obs}$ values were obtained by fitting equation 5 to integrals I of the ^{19}F resonance of the reporter at different relaxation times T with I_0 as integral at T = 0 s.

$$I = I_0 e^{-R_{2,obs}T} \qquad (5)$$

Fitted $R_{2,obs}$ values for each $CaCl_2$ concentration were used to fit EC_{50} values according to equation 1.

Cryogenic electron microscopy

Sample preparation. The DC-SIGN ECD sample in a buffer containing 25 mM HEPES, 150 mM NaCl, 10 mM CaCl2, pH 7.4 was thawed on ice and adjusted to a concentration of 0.02 mg mL^{-1} for vitrification. Subsequently, 3.5 µL were applied to glow-discharged holey carbon grids (Quantifoil R3/3, 300 mesh; Quantifoil Micro Tools GmbH, 1 min glow discharge), incubated for 45 s, blotted for 2 s, and plunge-frozen in liquid ethane using a Vitrobot IV device (FEI Company) operated at 4 °C and 90 % humidity.

Cryo-EM data acquisition and processing. Cryo-EM data were acquired at a FEI Tecnai G2 Polara microscope operating at 300 kV and equipped with a Gatan K2 Summit direct electron

detector in electron-counting mode. Images were recorded at 31,000x magnification (pixel size 0.625 Å) with a total dose of 60 $e^-/Å^2$. Automated data collection was carried out using Leginon with a defocus range of -1.5 to -2.5 µm.[9] Gain correction, dose-weighting, motion correction, and alignment of movie frames were performed using MotionCor2.[10]

Subsequent data processing was carried out with cryoSPARC v4.6.0 (Figure S5).[11] CTF estimation was performed with CTFFIND4.[12] After manual inspection of 3,574 micrographs, 3,398 micrographs were used for further analysis. Initial blob picking using a particle diameter of 350 Å and 2D classification yielded class averages that served as templates for picking particle with a diameter of 350 Å. Iterative 2D classification yielded 206,887 particles, which were extracted with a 768-pixel box size and Fourier-cropped to 192 pixels (2.5 Å/pixel). *Ab initio* reconstruction of particle images yielded two 3D reconstruction: one representing an artifactual structure (92,395 particles), and another resembling the extracellular domain of DC-SIGN (114,492 particles). The latter was subjected to a heterogeneous refinement, which removed 35,553 noisy particle images and improved the overall quality of the DC-SIGN ECD map, comprising 78,939 particles. Subsequent 2D classification and selection of high-quality 53,694 particle images, followed by *ab initio* reconstruction and non-uniform refinement, improved the definition of carbohydrate recognition domains and neck repeats in the DC-SIGN ECD structure.[13] Finally, particle images were re-extracted using an enlarged box size of 896 pixels, Fourier-cropped to 224 pixels (2.5 Å/pixel), and further refined through *ab initio* reconstruction as well as non-uniform refinement. This resulted in a final 3D reconstruction of DC-SIGN ECD, composed of 51,929 particle images (Figure 1J), with a low global resolution of approximately 7-8 Å, according to the Fourier shell correlation (FSC) 0.143 criterion (Figure S6).

To evaluate the cryo-EM map of DC-SIGN ECD, a 3D structural model was generated from the human DC-SIGN ECD sequence (UniProt ID: Q9NNX6) using AlphaFold3 (Figure S4).[14] The predicted atomic coordinates of DC-SIGN ECD were then rigid-body fitted into the cryo-EM map using UCSF ChimeraX.[15] Representative views of model-to-map fit are shown in Figure 1K, revealing differences in CRD arrangement and a reasonable fit of the neck repeats to the segmented region of the cryo-EM map. The cryo-EM map was deposited in the EMDB under accession EMD-56237.

Mixed solvent MD simulations

Simulation setup. The initial DC-SIGN coordinates were obtained from the PDB (PDB ID: 1SL4).[16] The carbohydrate ligand and crystallographic waters were removed, retaining only the three Ca^{2+} ions. The system was solvated in a truncated octahedral box with a 10 Å buffer using the OPC water model, and protein residues were parameterized with the AMBER ff19SB force field.[17] For mixed-solvent simulations, phenol was added to achieve a 5% v/v concentration. Initial energy minimization was carried out to relieve steric clashes and optimize the solvent configuration. The system was then gradually thermalized through a two-step protocol. The first step consisted of a 10 ps simulation under constant volume (NVT) conditions using a Berendsen thermostat. During this phase, the system was heated from 1 K to 10 K without the SHAKE algorithm, and positional restraints of 5 $kcal{\cdot}mol^{-1}{\cdot}Å^{-2}$ were applied to all heavy atoms of the protein to maintain structural integrity. Subsequently, an equilibration phase was performed in two stages. The first stage involved a 5 ns simulation under isothermal-isobaric (NPT) conditions (300 K, 1 atm) using a Langevin thermostat. Hydrogen bond constraints were maintained using the SHAKE algorithm, and weak harmonic restraints of 2 $kcal{\cdot}mol^{-1}{\cdot}Å^{-2}$ were applied to the protein backbone atoms. The integration time step was set to 1 fs. In the second stage, a 10 ns unrestrained equilibration was conducted under the same NPT conditions, utilizing the Langevin thermostat and SHAKE constraints, with an increased integration time step of 2 fs. Production simulations were then performed for a total duration of 1000 ns under NPT conditions at 300 K and 1 atm. Periodic boundary conditions were applied, and the SHAKE algorithm was used to constrain all bonds involving hydrogen atoms, allowing for a 2 fs integration time step. No positional restraints were imposed during the production runs.

To prevent the artificial aggregation of phenol molecules in the mixed solvent system, a dummy atom (DU) was introduced at the centroid of the aromatic ring of each phenol molecule. A repulsive Lennard-Jones potential was applied between these dummy atoms, with parameters $R_{min,i,j}$ = 6 Å and $\varepsilon_{i,i}$ = 0.01 $kcal{\cdot}mol^{-1}$, as described in previous implementations of Site Identification by Ligand Competitive Saturation (SILCS) methodology. All simulations were executed using the AMBER molecular dynamics package. Post-simulation analyses included root-mean-square deviation (RMSD) measurements, evaluation of protein-ligand interaction energies, and structural clustering to investigate conformational dynamics and binding site characteristics.

Determination of Water and Phenol Sites. High-occupancy regions for water and phenol were identified using Phenoltype, a VMD plugin (available upon request) based on the WatClust algorithm, with modifications to support clustering of any atom type from any cosolvent.[18] For Water Site (WS) identification, the positions of water oxygen atoms within 5 Å of the protein surface were extracted from successive MD snapshots. Atoms within 1.4 Å of each other were clustered, and the center of mass of each cluster was defined as the WS coordinate. The occupancy of each site was calculated as the probability of finding a water molecule within a 1 Å radius of the WS coordinate, normalized to bulk solvent density. For phenol, the clustering was performed using the DU atom as the reference. Phenoltype also calculates the number of molecules per cluster and their residence times. Only sites with occupancies exceeding 10% of the total simulation time were included in subsequent analyses. The FTMap webserver was used to compare solvent cluster of the MD-derived open state and the X-ray crystallographic structure of DC-SIGN (PDB ID: 1SL4).[19]

MD simulations - Apo vs Holo DC-SIGN

Simulation setup. A crystal structure of the DC-SIGN CRD (PDB ID: 1K9I) was prepared for simulation of the holo state by removal of ligand, crystal waters, and all but one protein chain.[20] To generate the apo state, the three Ca^{2+} ions were removed from the canonical binding sites. Using GROMACS 4.6, the systems were parameterized with the AMBER99SB-ILDN forcefield, solvated in TIP3P water, neutralized by addition of Cl^- ions, and energetically minimized using a steepest decent algorithm (emtol = 100 kJ/mol/nm, tau = 0.01).[21-23] Equilibrations of 100 ps in the NVT ensemble at 300 K using a Berendsen thermostat and of 150 ps in the NPT ensemble at 300 K/1 bar adding a Parrinello-Rahman barostat were carried out while putting position restraints on protein heavy atoms. Five random starting structures were selected from a 1 ns pre-run at 350 K to start one 400 ns production replica each (2000 ns total simulation time) writing solute coordinates to disk at dt = 1 ps. A leap-frog integrator was used with a 2 fs time step. Periodic boundary conditions were applied in x-, y-, and z-direction.

Mutual information analysis. Backbone and side chain dihedral angles were extracted from MD trajectories using MDTraj.[24] Normalized mutual information (NMI) values were calculated between all pairs of φ-ψ vs. φ-ψ (2D2D), φ-ψ vs. χ (2D1D), and χ vs. χ (1D1D) distributions using a custom Cython implementation.[25] The input distributions were discretized using 90 bins per

dimension (Figure S10). The obtained angle-wise NMI contributions were projected onto residue-wise contributions and not normalized but the resulting matrix was centered via

$$M_{ij} = M_{ij} - \frac{r_i \cdot c_j}{sum(M)},$$ with row sums r and column sums c.

Alignment and sequence analysis

Unique human structures of C-type lectins deposited in the PDB (38 total) were chosen for structural and sequence alignment using the PyMol and the MAFFT software.[26] Sequence logos were created using the WebLogo 3 software.[27]

Supplementary notes

Note S1: Mutant selection for biochemical assays

We initially selected eight point mutations based on insights from our MD simulations and NMR experiments and expressed their CRDs. All mutant holo ^{15}N-labeled CRDs produced well-dispersed spectra indicating correct folding of the proteins (Figure S11). Superposition of the mutant to the wildtype spectra revealed large CSPs in residues in close proximity to and over 10 Å away from the mutation sites. These remote, non-nearest neighbor effects were most pronounced for the T314A, E358A, F359L and W364F mutants, suggesting mutation of hub residues to globally affect the CRD fold (Figure S12). In mannose titrations, all mutant CRDs showed characteristic CSPs upon addition of mannose, further confirming correct folding and activity of the proteins (Figure S13). Nevertheless, fitting of K_Ds revealed significant reduction in affinity for mutations E358A and F359L and to a lesser extent for mutants F313A and W364F at the extended CBS (Figure S14 and S15). To avoid direct interference with the canonical CBS and Ca^{2+} cage in subsequent assays, we selected T314A, a key hub residue identified in MD simulations that induced long-range CSPs while only marginally affecting mannose affinity.

Note S2: Retrospective analysis of X-ray structures suggests a stabilizing effect of ligand binding at the cryptic pocket

To date, no high affinity ligand or co-crystal structure of a ligand-occupied cryptic site is available. Yet we reasoned that changes in stability and conformation should be reflected in the comparison of the X-ray structures of wildtype CRD in the open and closed conformation. Comparison of the normalized B factors in wildtype CRD X-ray structures in the open (PDB ID: 1SL5) and closed

(PDB ID: 1SL4) conformations revealed that pocket opening alters structural flexibility (Figure S25A).[16] Normalized B factors decreased at the N-terminal end of $\alpha 2$ but increased at its C-terminal end, forming the upper cavity. While the latter could result from the M270 sidechain competing water from the upper cavity, decreased normalized B factors of residues in $\alpha 2$ suggest increased stability upon pocket opening and binding of the tryptophan sidechain at the cryptic pocket (Figure S25B and S4). Similarly, decreased B factors in the loop and $\beta 3$ and 4 suggest stabilization at the extended carbohydrate binding site.

Overall, changes in stability were not accompanied by major backbone rearrangements. However, in accordance with our phenol-MD simulations, water competition in the upper cavity altered the hydrogen bonding pattern of T314 (Figure S1B). In the closed state, T314 forms a hydrogen bond to a water molecule, while rotation of M270 in the open state removes water, enabling a T314-S307 hydrogen bond in $\alpha 2$ (Figure S25C). Despite the flexibility of the S307 sidechain, this may stabilize interactions between $\alpha 2$ and $\beta 2$ and influences the adjacent $\alpha 2$-$\beta 2$ loop bearing residue F313 involved in oligosaccharide binding. Our apo-holo MD simulations showed this bond to occur exclusively in the holo state while being absent in the apo state (Table S1). Consequently, the T314A mutant likely reduces water occupancy in the upper cavity, allowing M270 rotation and favoring the open conformation even in the absence of a ligand. Moreover, it precludes the T314-S307 hydrogen bond, which may also destabilize the holo state, trapping $\alpha 2$ in its apo conformation. Although no apo X-ray structure exists, our MD simulations also suggested a hydrogen bond between Q300 ($\alpha 2$) and W364 ($\beta 4$, WND motif) to be weakened in the apo state. The WND motif links Ca^{2+} coordination at the glycan-binding site to the hydrophobic core and, as our data suggest, also to $\alpha 2$.[28]

Supplementary figures

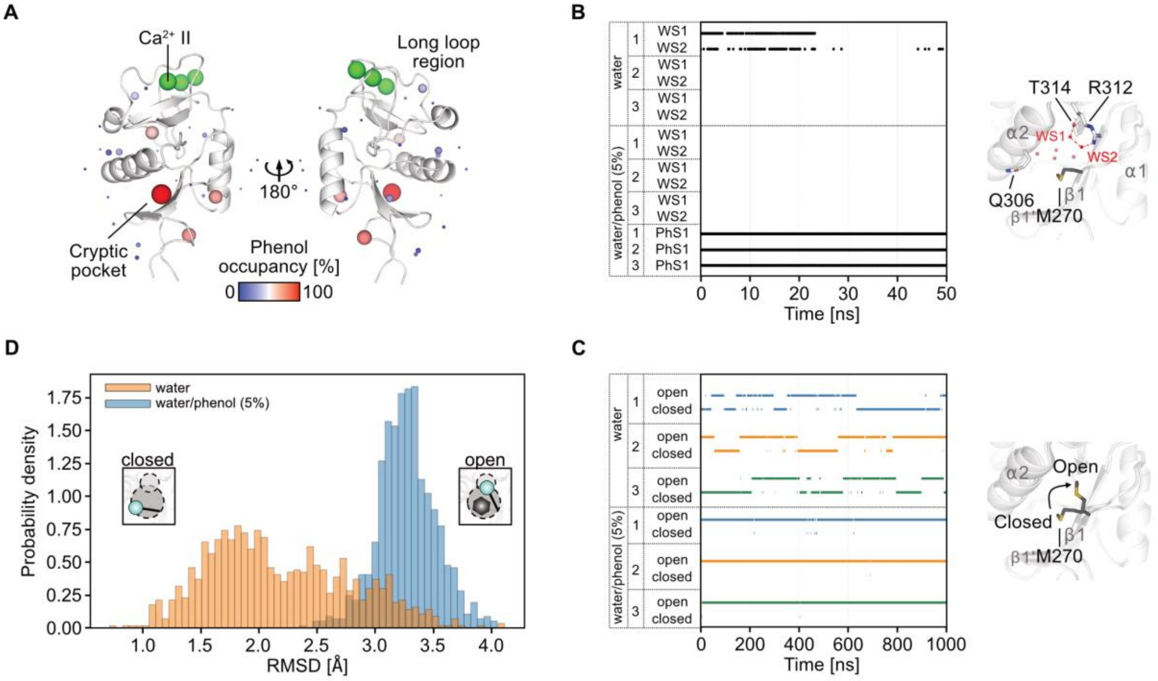

Figure S1. Mixed solvent MD simulations with phenol uncover a cryptic pocket. (**A**) Phenol occupancy clusters on the DC-SIGN CRD surface over the course of the simulations. Twenty-seven clusters appearing in ≥10% of the total simulation time are shown as spheres, scaled and colored by relative occupancy. The cryptic pocket is identified as the most frequently occupied site. (**B**) Solvent structure analysis of the cryptic pocket during the final 50 ns of each 1 µs simulation replicate 1-3. Hotspots for water molecules WS1 and WS2, located in the upper cavity near T314 and R312, can be displaced by M270 side-chain rotation in both water (intermittently) and 5% phenol simulations (persistently). When phenol is present at the phenol hotspot representing the cryptic pocket (PhS1), it occupies the cryptic pocket and stabilizes the open conformation, preventing reformation of WS1 and WS2. (**C**) Cluster-based time-series analysis of the conformation of residue M270 relative to its conformation in PDB ID: 1SL4. All frames across the full simulation time with a side chain RMSD ≤ 2 Å were labeled as "closed," while those with RMSD ≥ 2.5 Å were tagged as "open". Intermediate values were considered negligible for this specific analysis. In water, M270 alternates between open and closed conformations, while in phenol-containing simulations, phenol binding stabilizes the open state for the majority of the trajectory. (**D**) Distribution of M270 side chain RMSD values relative to the closed conformation across all frames and replicates. The probability density plot summarizes the same data used in (C), showing a bimodal distribution in water (orange) and a shift toward the open state in 5% phenol (blue). RMSD values were binned and normalized to produce probability densities, highlighting the phenol-induced stabilization of the open conformation.

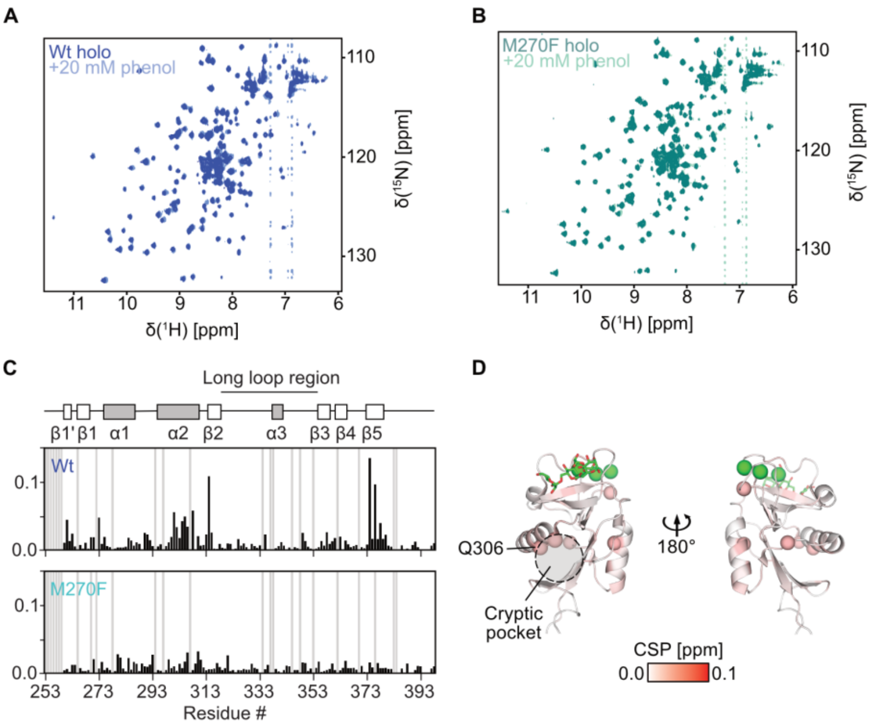

Figure S2. The M270F mutation blocks DC-SIGN from interacting with phenol. (**A**) and (**B**) ^1H-^{15}N HSQC NMR spectra of holo DC-SIGN CRD wildtype and M270F in the absence or presence of phenol. (**C**) Comparison of CSPs maps of the wildtype and M270F interacting with 20 mM phenol, show drastically reduced CSPs for the mutant. (**D**) Mapping of CSPs observed addition of 20 mM phenol to holo DC-SIGN CRD M270F confirm inhibition of phenol binding by the mutant. Ca atoms of residues experiencing a CSP > 0.025 ppm are shown as spheres.

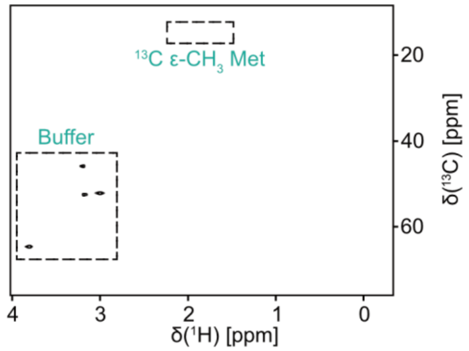

Figure S3. ^{1}**H-**^{13}**C HSQC NMR spectrum without** ^{13}**C ε-methyl methionine labeled DC-SIGN CRD wildtype**. Spectra of samples containing only buffer (20 mM MES, 40 mM NaCl, 10 mM CaCl2, pH 6.0) without labeled protein served to locate buffer peaks and exclude scrambling of the ^{13}C ε-methyl methionine during protein expression.

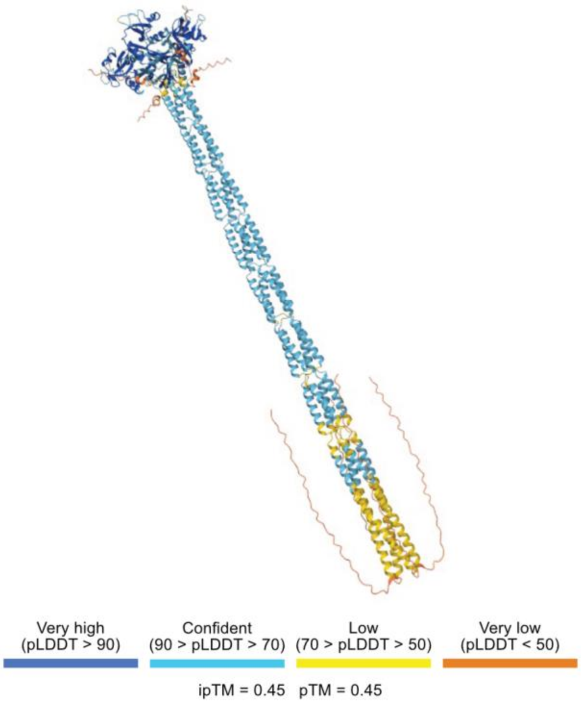

Figure S4. AlphaFold3-predicted structure of the human DC-SIGN ECD. The model was generated using the protein sequence of human DC-SIGN ECD (Uniprot ID: Q9NNX6). Confidence levels of the predicted structure are color-coded as indicated by the scale bar.

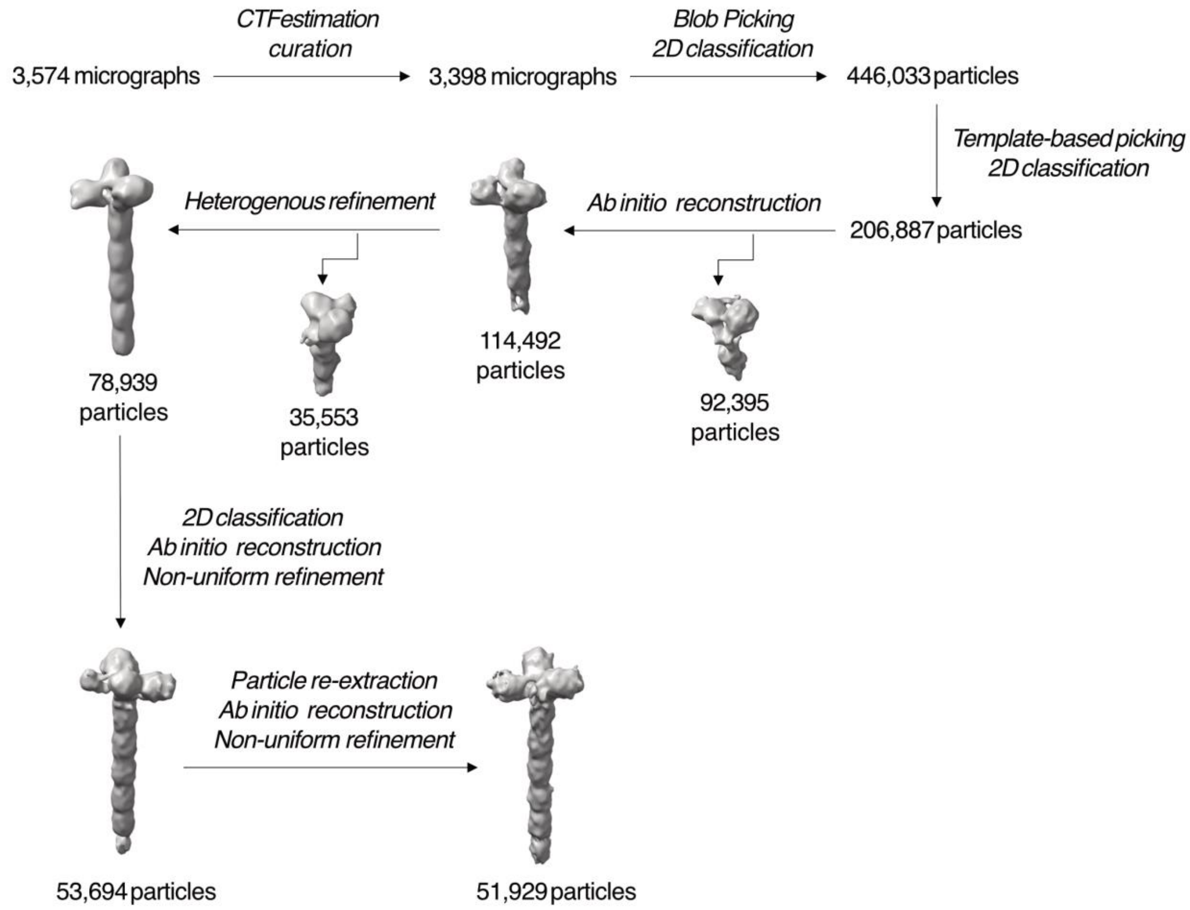

Figure S5. Cryo-EM refinement sorting scheme of the DC-SIGN ECD dataset. After CTF estimation and manual micrograph inspection, initial blob picking and 2D classification were performed. The resulting 2D class averages served as templates for particle picking. Iterative 2D classification yielded 206,887 particle images, which were extracted with a box size of 768 pixels and subsequently Fourier-cropped to 192 pixels (2.5 Å/pixel). *Ab initio* reconstruction of these particle images revealed a 3D reconstruction resembling the expected structure of DC-SIGN ECD. This 3D reconstruction was subjected to heterogeneous refinement, which removed noisy particles and improved the overall DC-SIGN ECD structure, consisting of 78,939 particle images. Subsequent 2D classification and selection of high-quality particle images, followed by *ab initio* reconstruction and non-uniform refinement, improved the definition of the CRDs and neck repeats in the DC-SIGN ECD structure. Finally, these particle images were then re-extracted with an increased box size of 896 pixels, Fourier-cropped to a box size of 224 pixels (2.5 Å/pixel) and subjected once more to *ab initio* reconstruction and non-uniform refinement. This resulted in a final 3D reconstruction of DC-SIGN ECD at a global resolution of 7-8 Å (FSC 0.143 criterion).

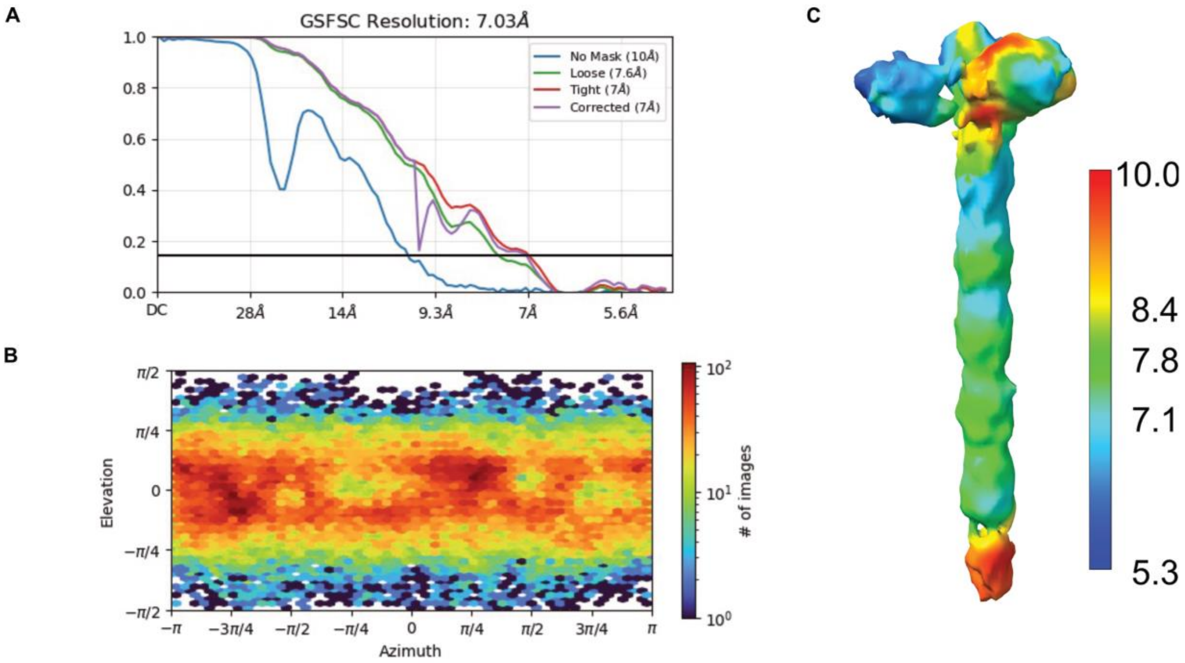

Figure S6. Global resolution estimation of cryo EM maps of DC-SIGN ECD. (**A**) The global resolution estimation, according to the FSC 0.143 criterion, was performed in cryoSPARC. (**B**) Projection direction distribution from non-uniform refinement in cryoSPARC, showing a preference for side views and underrepresentation of top views. (**C**) Final cryo-EM map colored by its local resolution estimation ranging from 5.3 to 10.0 Å as indicated by the colored scale bar.

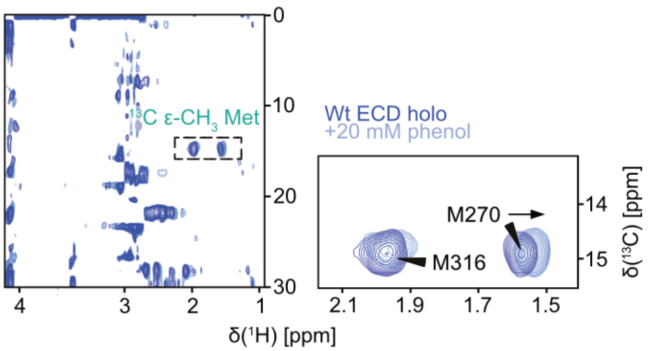

Figure S7. The cryptic pocket is accessible in DC-SIGN ECD in solution. ^{1}H-13C TROSY NMR spectra of ^{13}C Met-labeled DC-SIGN ECD in the presence or absence of 20 mM phenol. The M270 resonance shows a clear shift similar to what is observed in the CRD (Figure 1G), indicating that the cryptic pocket is accessible in the tetrameric ECD.

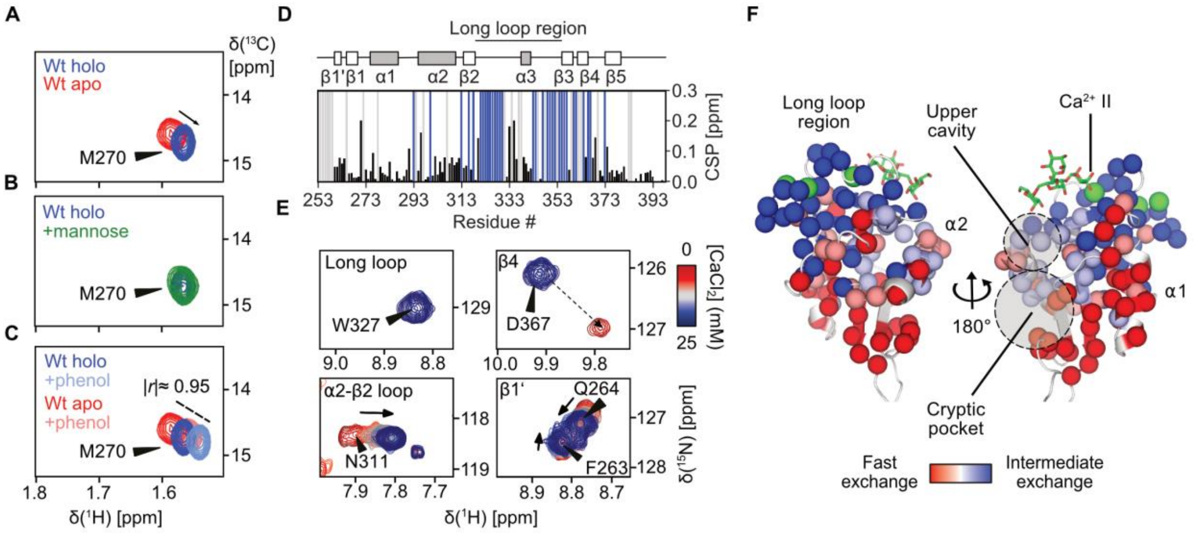

Figure S8. Ca^{2+} binding induces conformational change throughout the CRD. (**A**) Removal of Ca^{2+} from ^{13}C ε-methyl methionine-labeled DC-SIGN CRD shifts the resonance of M270 indicating conformational change at the cryptic site. (**B**) Addition of 20 mM mannose to holo DC-SIGN CRD does not induce shift in M270. (**C**) Addition of 20 mM phenol to apo DC-SIGN shifts M270 along the same trajectory towards the holo state. Pearson correlation of the chemical shift position indicates linear correlation of the holo, and the phenol bound state. (**D**) CSPs induced by addition of 25 mM $CaCl_2$ to ^{15}N-labeled apo DC-SIGN CRD wildtype in ^{1}H-^{15}N HSQC NMR experiments reveals global conformational change throughout the CRD. (**E**) Residues in different regions of the CRD show different exchange regimes upon titration of Ca^{2+}. (**F**) Qualitative mapping of exchange regimes as observed in Ca^{2+} titrations reveal α2 to be in fast-to-intermediate exchange, while the long loop region is in intermediate exchange and more distal regions, including the cryptic site, are in fast exchange.

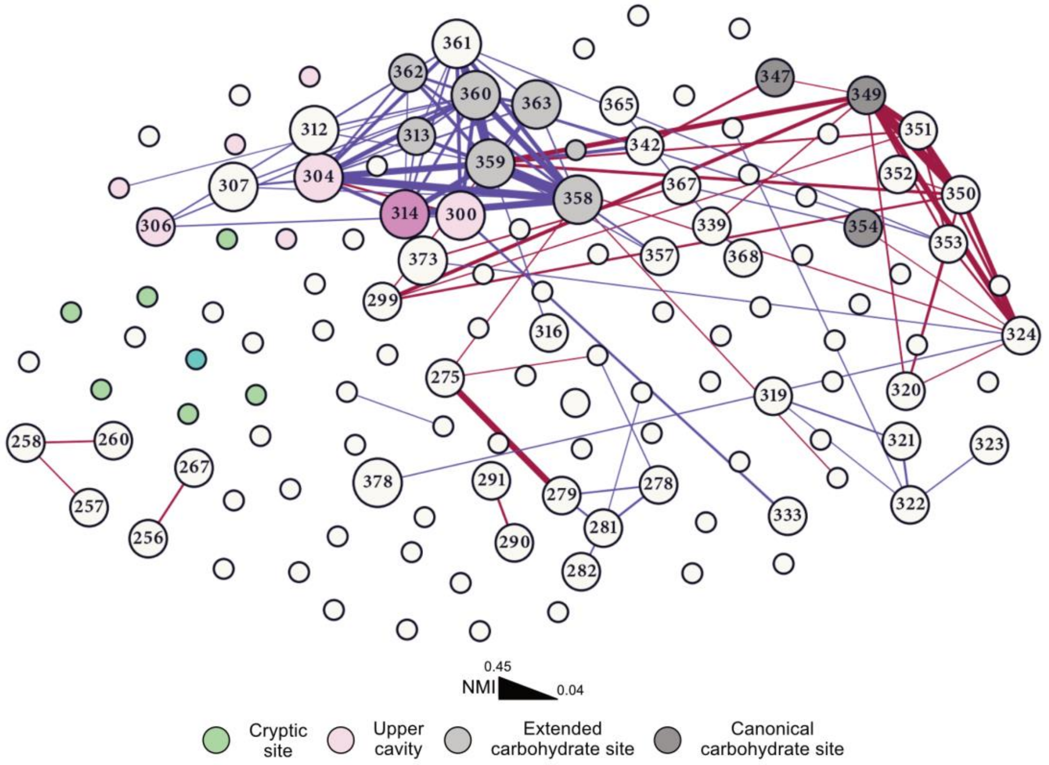

Figure S9. Ca^{2+} binding shifts the connectivity of the DC-SIGN CRD. Network representation of the difference in NMI values computed for the holo and apo state (holo - apo) of DC-SIGN CRD. Blue and red edges illustrate higher NMI values in the holo and apo state, respectively. Scaling and coloring of nodes and edges as described for figure 2A.

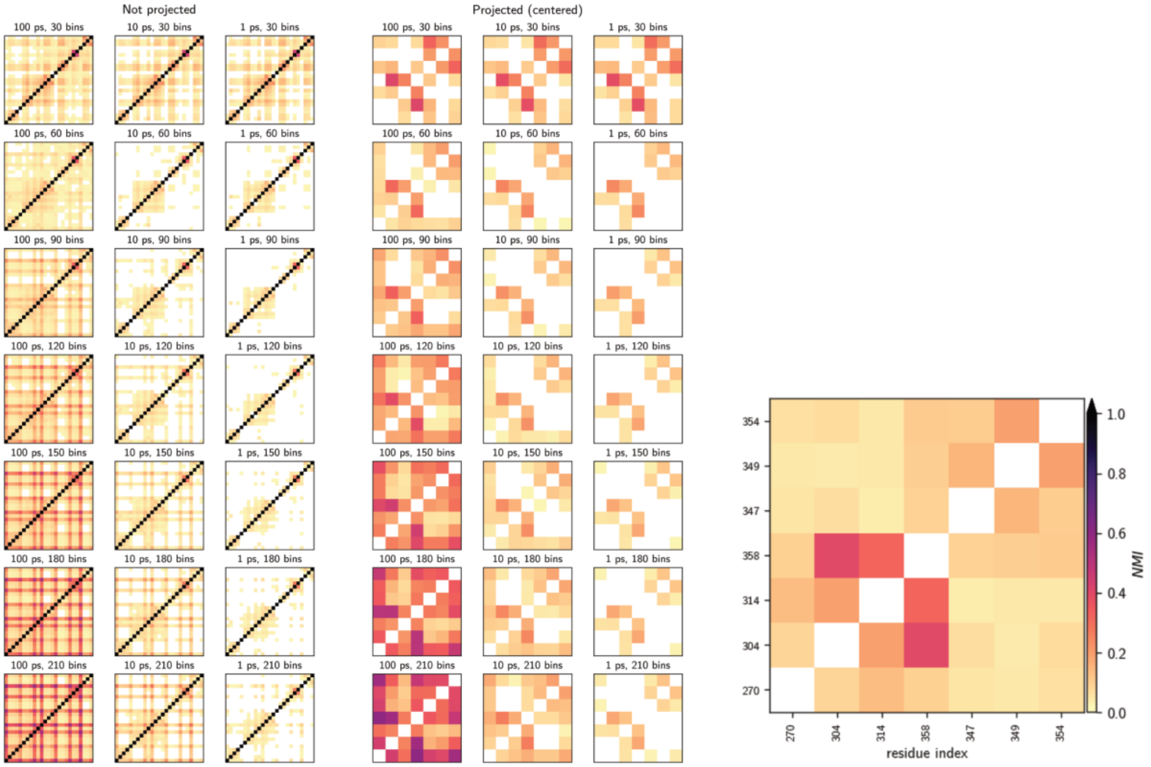

Figure S10. Effect of striding and binning on computed NMI values. For all pairs of dihedral angle distributions in residues 270, 304, 314, 347,349, 354, and 358 we screened trajectory strides of 1, 10, and 100 ps in combination with 30 to 210 histogram bins when computing NMI values and plotted the original (left) and residue-wise projected (right) NMI matrices. In order to avoid poor resolution as well as noisy estimates, we settled on using 1 ps/90 bins for the full analysis.

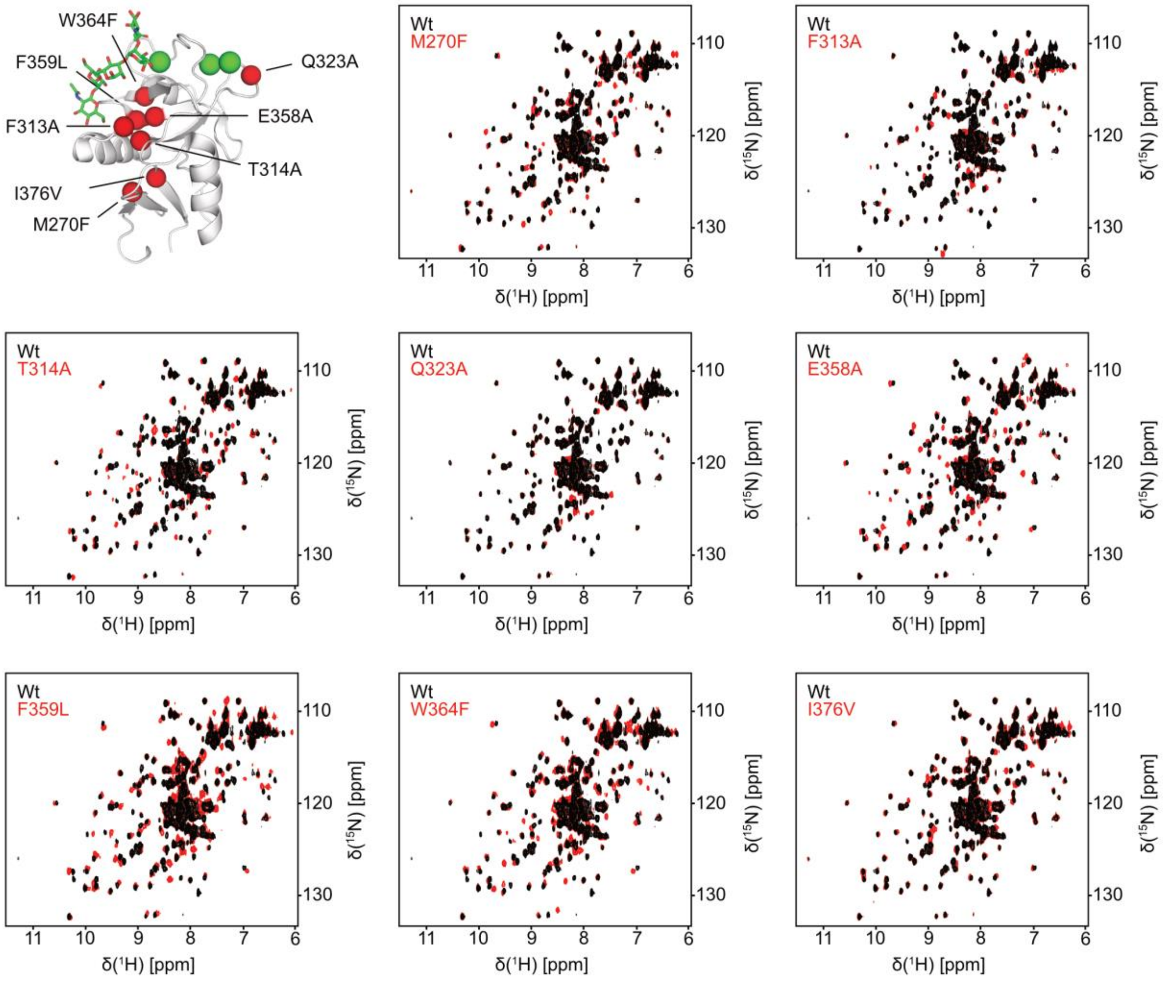

Figure S11. Point mutations in DC-SIGN yield folded CRDs. Locations of point mutation mapped to the X-ray crystallographic structure of DC-SIGN CRD (PDB ID: 1K9I). Red spheres indicate Ca atoms of positions selected for mutation in the DC-SIGN CRD. ^{1}H-^{15}N HSQC NMR spectra of holo DC-SIGN CRD wildtype and mutants show dispersed spectra with significant chemical shift differences to the wildtype protein.

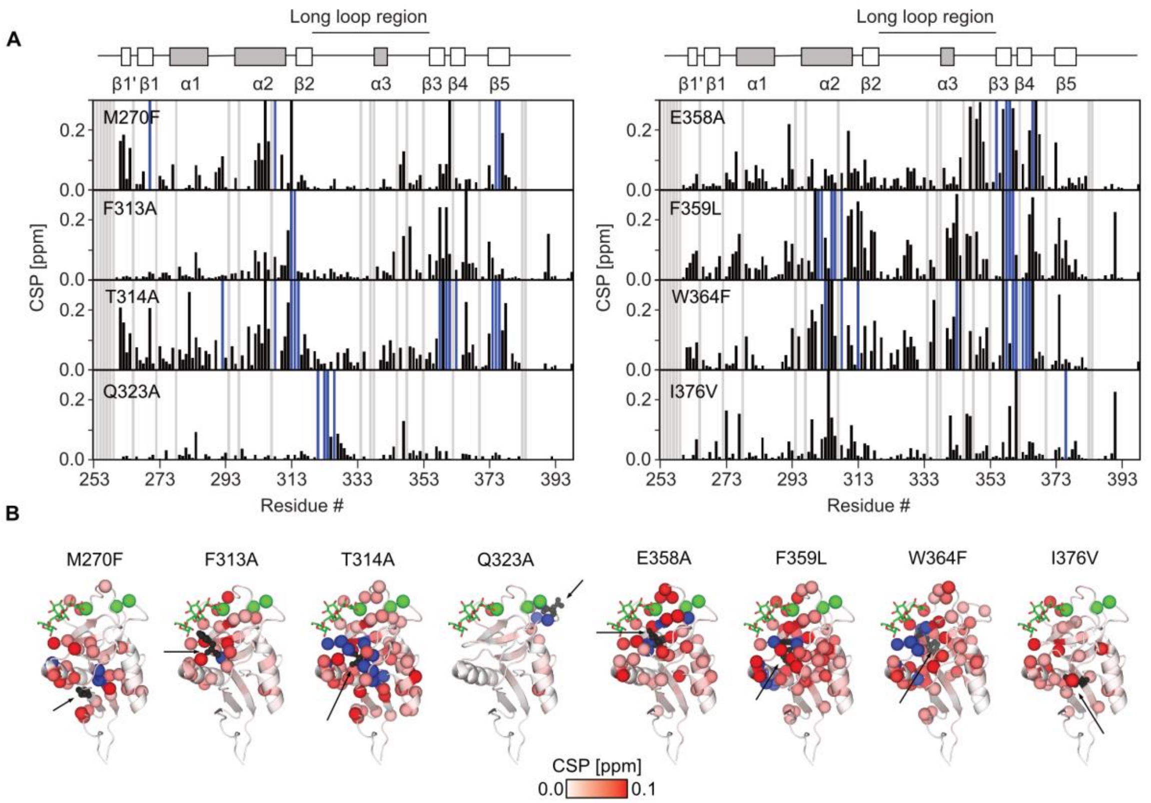

Figure S12. Mutation of hub residues induce chemical shift perturbations distal to the mutation site. (**A**) CSP maps illustrating chemical shift changes induced by point mutations relative to the wildtype in ^{1}H-^{15}N HSQC NMR spectra. Grey bars indicate unassigned residues. Blue bars indicate resonances of residues that experience severe line broadening or where assignment could not be unambiguously transferred due to large CSPs. (**B**) CSPs induced by point mutations mapped to the structure of DC-SIGN CRD. Ca atoms of residues experiencing a CSP > 0.025 ppm are shown as spheres. Blue spheres indicate resonances of residues that experience severe line broadening or where assignment could not be unambiguously transferred due to large CSPs. Mutated residues are shown as black spheres.

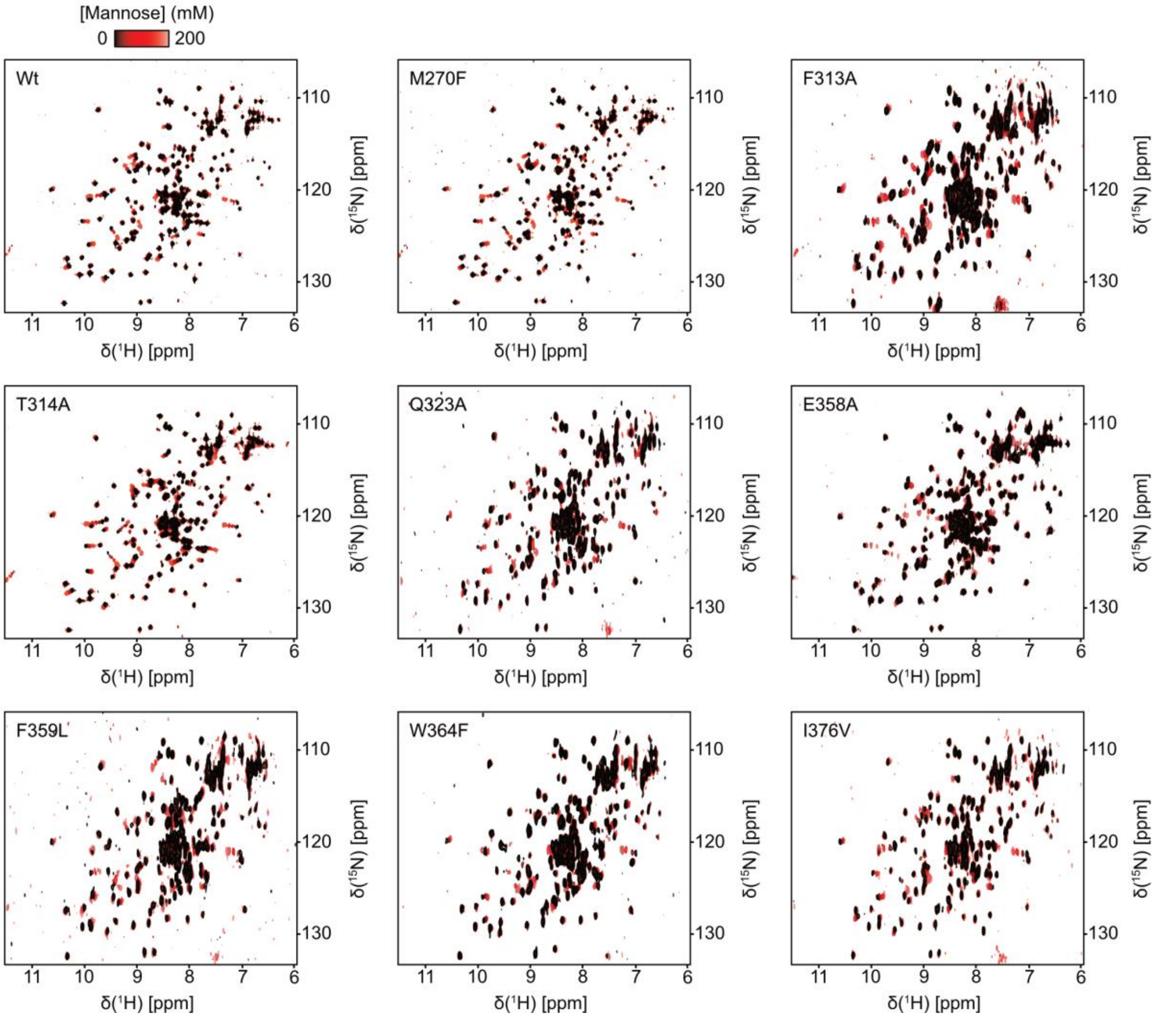

Figure S13. The mutant CRDs interacting with mannose. ^{1}H-^{15}N HSQC (Wt, M270F, T314A, E358A) and SOFAST-HMQC (F313A, Q323A, F359L, W364F, I376V) NMR spectra of holo DC-SIGN CRD wildtype and mutants at varying concentrations of mannose. All proteins show characteristic CSPs upon addition of mannose, indicating correct folding and functionality of the CRDs.

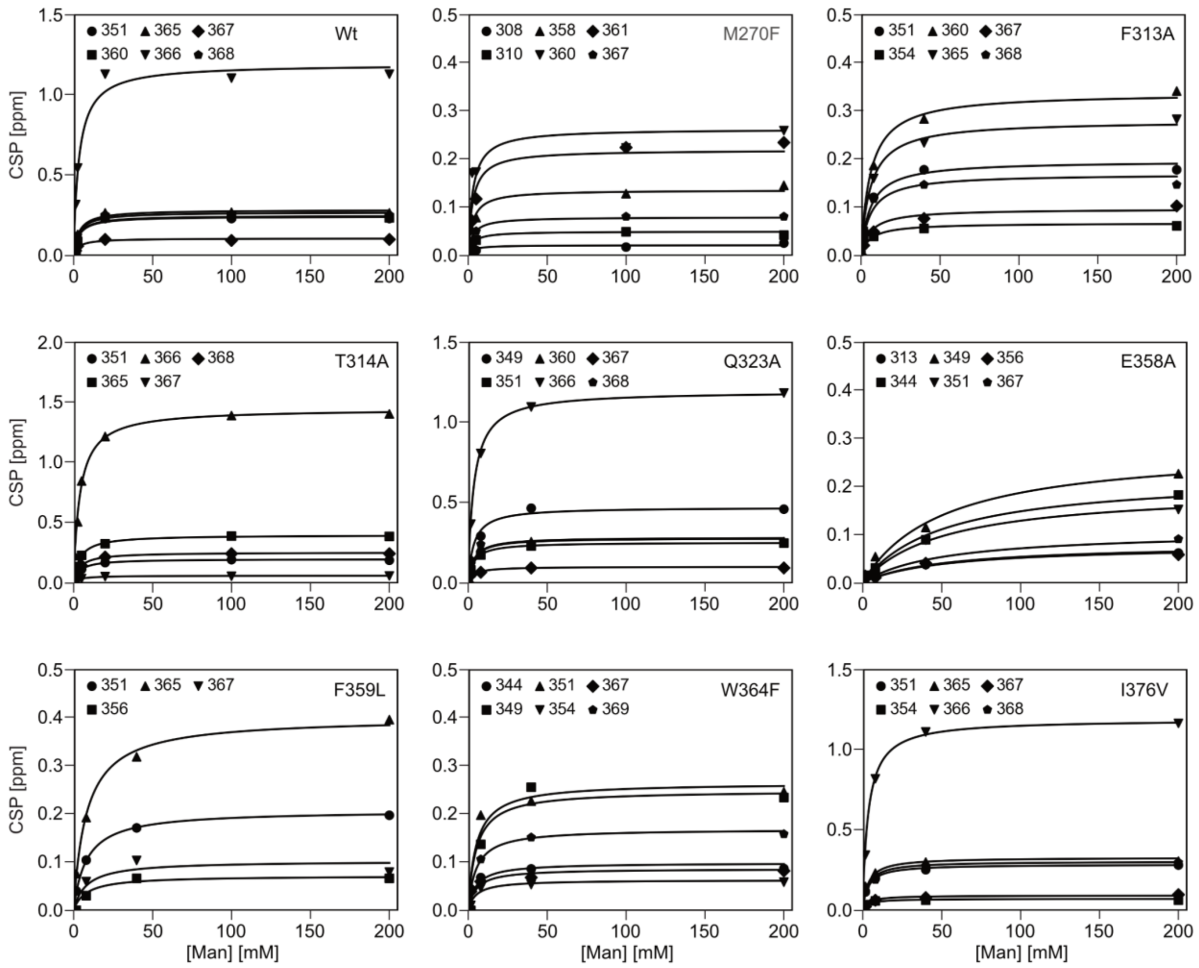

Figure S14. Affinities of wildtype and mutant CRDs interacting with mannose. Global fitting of CSP trajectories reveals similar mannose affinities for the wildtype (K_D = 3.3 ± 0.3 mM) and for the M270F (K_D = 2.9 ± 0.4 mM), T314A (K_D = 3.9 ± 0.1 mM), Q323A (K_D = 3.5 ± 0.2 mM), and I376V (K_D = 3.4 ± 0.2 mM) mutants, while mutations of hub residues F313A (K_D = 6.0 ± 0.5 mM), E358A (K_D = 51.1 ± 4.9 mM), F359L (K_D = 8.2 ± 0.8 mM) and W364F (K_D = 5.0 ± 0.9 mM) show reduced affinity.

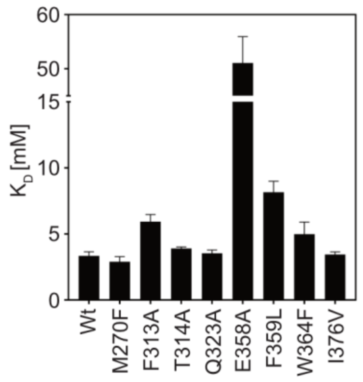

Figure S15. Mutation of hub residues differentially modulate mannose affinity. Comparison of fitted mannose affinities from ^{1}H-^{15}N HSQC NMR titrations (see also Figure S14).

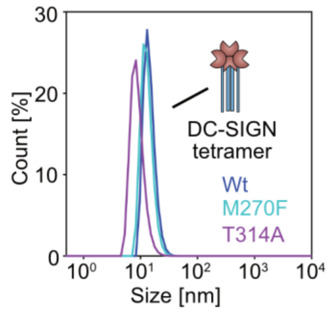

Figure S16. The mutant proteins form oligomers in solution. DLS measurements of the ECD proteins in 25 mM HEPES, 150 mM NaCl, 10 mM CaCl2, pH 7.4. Inflection points reveal no significant changes in tetramerization properties.

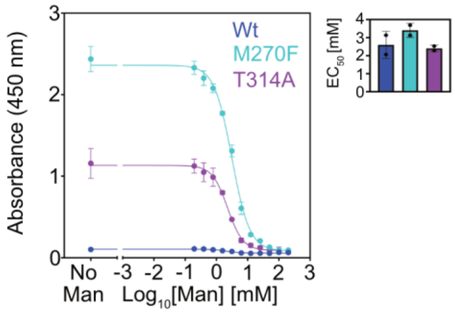

Figure S17. Inhibition of HRP binding by mannose. Binding of HRP at constant concentration to the ECD proteins at varying mannose concentrations, reveal marginal changes in the mannose IC_{50} for the M270F (IC_{50} = 3.1 mM, hill slope = -1.5) and T314A (IC_{50} = 2.1 mM, hill slope = -2.1) mutations compared to the wildtype (IC_{50} = 2.3 mM, hill slope = -1.8).

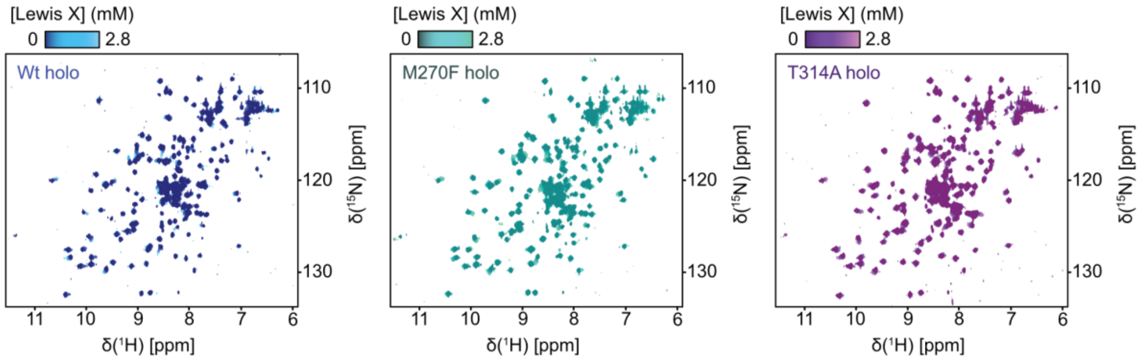

Figure S18. The mutant CRDs interacting Lewis X. ^{1}H-^{15}N HSQC NMR spectra of holo DC-SIGN CRD wildtype and mutants at varying concentrations of the Lewis X trisaccharide. All proteins show characteristic CSPs upon addition of Lewis X.

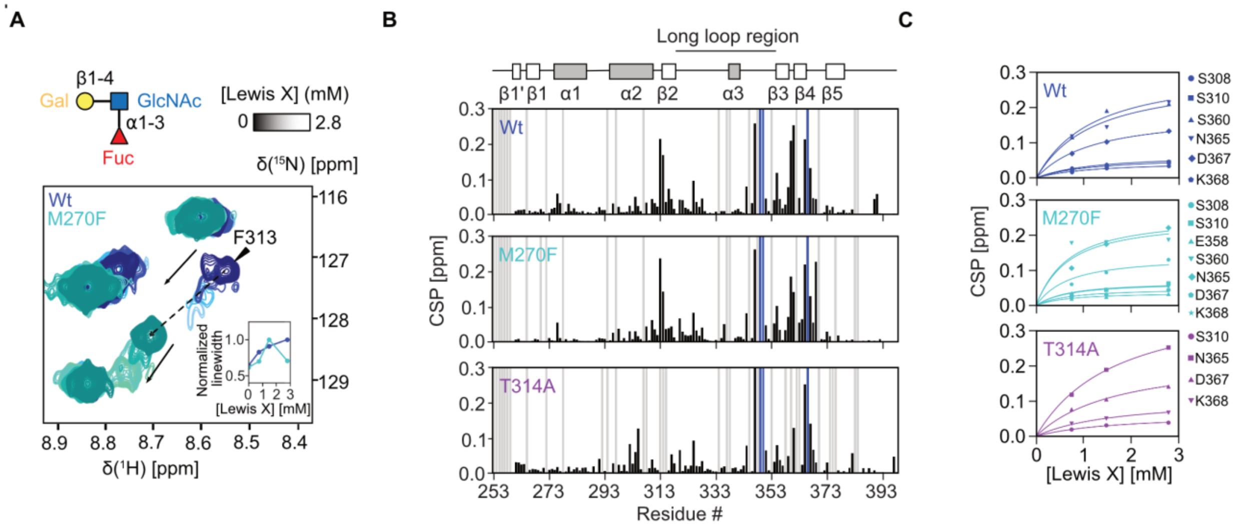

Figure S19. The mutant CRDs bind Lewis X. (**A**) Comparison of ^{1}H-^{15}N HSQC NMR shift trajectory of residue F313 upon titration of the Lewis X oligosaccharide to wildtype and M270F CRD. For both proteins, F313 is in intermediate exchange but faster recovery of the linewidth indicates M270F to saturate faster than the wildtype protein. (**B**) CSP maps of 3 mM Lewis X interacting with wildtype and the mutant CRDs. (**C**) Fitting of CSP trajectories reveals M270F (K_D = 0.6 ± 0.2 mM) to increase affinity for Lewis X while T314A (K_D = 1.4 ± 0.1 mM) has decreased affinity for Lewis X compared to the wildtype CRD (K_D = 1.1 ± 0.2 mM).

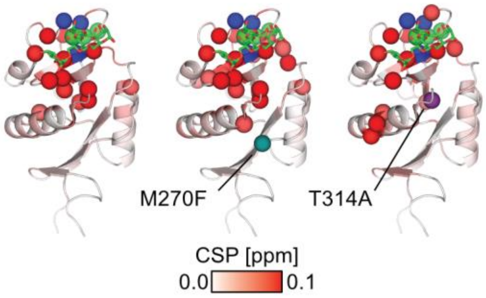

Figure S20. Lewis X induces CSPs at the canonical and extended carbohydrate binding site. Mapping of CSPs induced by the addition of 2.8 mM Lewis X to the structure of DC-SIGN CRD (PDB ID: 1SL4), suggest the ligand to interact with the canonical and the extended carbohydrate binding site. T314A shows additional CSPs in α2. Ca atoms of residues experiencing a CSP > 0.025 ppm are shown as spheres. Blue spheres indicate resonances of residues that experience severe line broadening or where assignment could not be unambiguously transferred due to large CSPs. M270F and T314A mutation sites are shown as dark cyan and purple spheres, respectively.

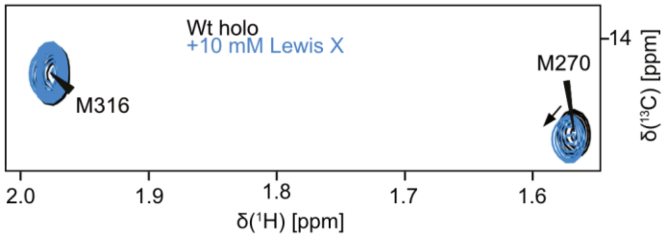

Figure S21. Lewis X binding affects the conformation of the cryptic site. ^{1}H-^{13}C HSQC NMR spectra of holo ^{13}C Met-labeled DC-SIGN CRD wildtype interacting with Lewis X shows a shift in the resonance of M270, while the resonance of residue M316 remains unchanged.

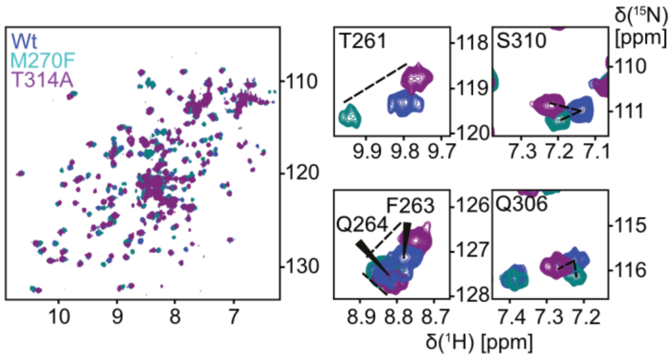

Figure S22. Mutations M270F and T314A induce linear and non-linear chemical shift changes. Comparison of ^{1}H-^{15}N HSQC NMR spectra of holo DC-SIGN CRD wildtype, M270F and T314A. Resonances of residues T261, F263 and Q264 are shown as example for linear displacement of chemical shifts upon mutation. Resonances of residues S310 and Q306 are shown as examples for non-linear behavior.

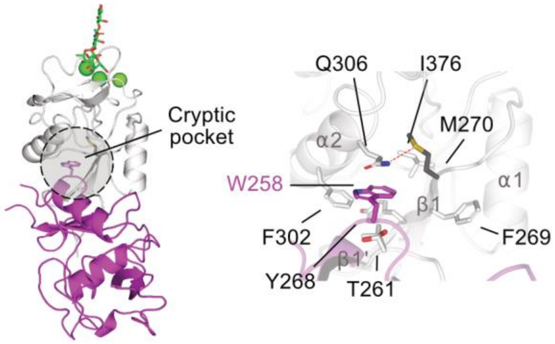

Figure S23. The open cryptic site in the X-ray crystallographic structure of DC-SIGN CRD wildtype. Residue W258 from a symmetry mate (pink cartoon) in the X-ray crystallographic structure of DC-SIGN CRD in complex with lacto-N-fucopentaose III (white cartoon, PDB: 1SL5) opens the cryptic pocket. M270 rotates into the upper cavity and is additionally stabilized by a predicted hydrogen bond (red dotted line) to residue Q306 in α2.

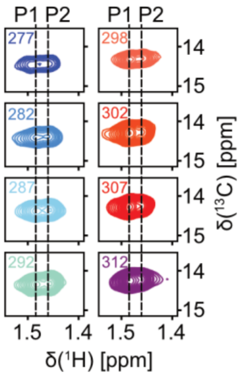

Figure S24. Temperature-dependent chemical shift changes of the M270 resonance in apo DC-SIGN T314A. ^{1}H-^{13}C HSQC NMR spectra of ^{13}C ε-methyl methionine-labeled apo DC-SIGN CRD T314A at different temperatures. Only the resonance of M270 is shown. With increasing temperature, state P1 becomes more populated, indicating an entropically driven transition from the holo-like state P2. See also figure 5H,I.

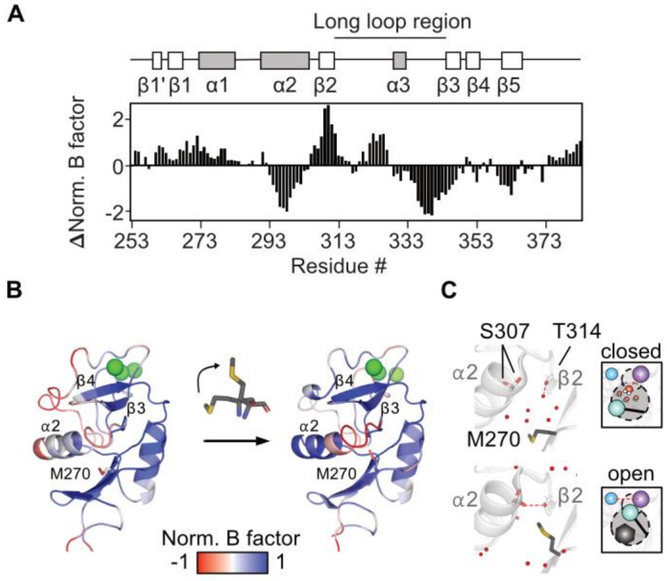

Figure S25. Ligand binding at the cryptic pocket stabilizes the CRD. (**A**) Difference of normalized B factors extracted from Ca atoms in the X-ray crystallographic structures of DC-SIGN CRD in the closed (PDB ID: 1SL4) and in the open state (PDB ID: 1SL5). Lower values indicate residues with higher B factor in the closed form and vice versa. (**B**) Normalized B factors extracted from all atoms of the same structures and mapped back onto the structures display changes in stability around α2 and β3 and 4. (**C**) Close up of the upper cavity in the closed (top, PDB ID: 1SL4) and the open form (bottom, PDB ID: 1SL5) displaying changes in the hydrogen bond pattern upon opening of the cryptic pocket.

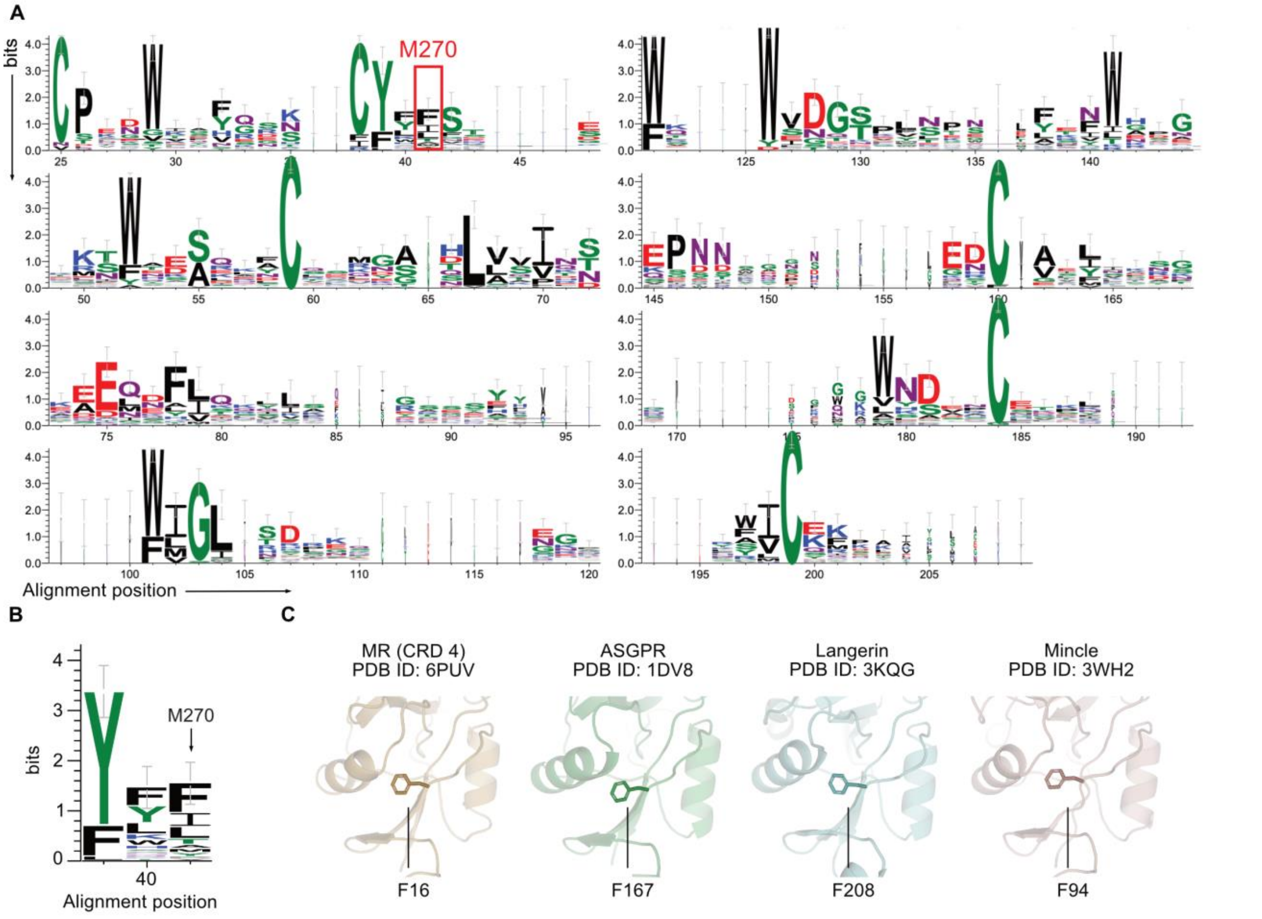

Figure S26. Phenylalanine is evolutionary preferred in position of M270. (**A**) Amino acid sequence logo generated from an alignment of 38 human C-type lectin domain structures. The alignment position 41 corresponding M270 in DC-SIGN is highlighted in red. Letter height denotes information content. Alignment positions before position 25 showed low conservation and were left out for clarity. (**B**) Inset of sequence logo, showing phenylalanine as the preferred amino acid in position of M270. (**C**) Example C-type lectins expressing phenylalanine in position of M270.

Supplementary tables

Table S1. Selected hydrogen bond populations of apo and holo DC-SIGN.

		holo	apo	
**Donor[a]**	**Acceptor[a]**	**Occupancy**	**Occupancy**	**ΔHB[b]**
N322s	T326m	18.57%	10.00%	8.57%
S360m	G363m	15.85%	9.80%	6.05%
N344s	S360m	5.84%		5.84%
S360s	E358s	4.79%		4.79%
V292m	I376m	37.73%	34.19%	3.54%
K379s	V287m	46.35%	43.10%	3.25%
T314s	S307s	3.25%		3.25%
F374m	F313m	2.95%		2.95%
W315m	F374m	75.49%	72.65%	2.84%
A382m	P255m	21.82%	19.04%	2.78%
D320m	D355m	25.92%	23.41%	2.51%
T261m	Y268m	45.24%	42.85%	2.39%
G363m	S360m	2.39%		2.39%
R345m	N344s	2.29%		2.29%
N266m	F263m	21.73%	19.52%	2.21%
Y342m	F339m	5.96%	3.85%	2.11%
R275s	D279m	40.64%	38.55%	2.09%
R275s	D279s	30.87%	28.79%	2.08%
R345s	N344s	1.09%	3.10%	-2.01%
K379m	C267m	36.90%	39.14%	-2.24%
N362m	S360s	9.82%	12.28%	-2.46%
F302m	E298m	34.03%	36.49%	-2.46%
N350m	D366s	67.66%	70.30%	-2.64%
F359m	T314m	80.85%	83.53%	-2.68%
S307s	L303m	61.38%	64.08%	-2.70%
Q341m	Q341s	12.04%	14.90%	-2.86%
S308s	Q304m	36.55%	39.59%	-3.04%
Q304s	G361m	32.29%	35.72%	-3.43%
T314m	F359m	26.58%	30.05%	-3.47%
Q300s	Q304s	22.93%	27.14%	-4.21%
S319s	D355m	15.67%	20.32%	-4.65%
W364s	Q300s	40.17%	44.98%	-4.81%

[a] s = sidechain, m = main chain

[b] Difference between populations of hydrogen bonds observed in simulations of holo - apo state of DC-SIGN CRD.

Supplementary references

(1) Wawrzinek, R.; Wamhoff, E.-C.; Lefebre, J.; Rentzsch, M.; Bachem, G.; Domeniconi, G.; Schulze, J.; Fuchsberger, F. F.; Zhang, H.; Modenutti, C.; et al. A Remote Secondary Binding Pocket Promotes Heteromultivalent Targeting of DC-SIGN. *Journal of the American Chemical Society* **2021**, *143* (45), 18977-18988. DOI: 10.1021/jacs.1c07235 From DOI.org (Crossref).

(2) Vranken, W. F.; Boucher, W.; Stevens, T. J.; Fogh, R. H.; Pajon, A.; Llinas, M.; Ulrich, E. L.; Markley, J. L.; Ionides, J.; Laue, E. D. The CCPN data model for NMR spectroscopy: Development of a software pipeline. *Proteins: Structure, Function, and Bioinformatics* **2005**, *59* (4), 687-696. DOI: 10.1002/prot.20449.

(3) Liu, M.; Mao, X.-a.; Ye, C.; Huang, H.; Nicholson, J. K.; Lindon, J. C. Improved WATERGATE Pulse Sequences for Solvent Suppression in NMR Spectroscopy. *J Magn Reson* **1998**, *132* (1), 125-129. DOI: 10.1006/jmre.1998.1405.

(4) Mori, S.; Abeygunawardana, C.; Johnson, M. O.; van Zijl, P. C. Improved sensitivity of HSQC spectra of exchanging protons at short interscan delays using a new fast HSQC (FHSQC) detection scheme that avoids water saturation. *J Magn Reson B* **1995**, *108* (1), 94-98. DOI: 10.1006/jmrb.1995.1109 From NLM Medline.

(5) Pederson, K.; Mitchell, D. A.; Prestegard, J. H. Structural Characterization of the DC-SIGN–LewisX Complex. *Biochemistry* **2014**, *53* (35), 5700-5709. DOI: 10.1021/bi5005014.

(6) Williamson, M. P. Using chemical shift perturbation to characterise ligand binding. *Prog Nucl Magn Reson Spectrosc* **2013**, *73*, 1-16. DOI: 10.1016/j.pnmrs.2013.02.001 From NLM Medline.

(7) Selvaratnam, R.; VanSchouwen, B.; Fogolari, F.; Mazhab-Jafari, Mohammad T.; Das, R.; Melacini, G. The Projection Analysis of NMR Chemical Shifts Reveals Extended EPAC Autoinhibition Determinants. *Biophysical Journal* **2012**, *102* (3), 630-639. DOI: 10.1016/j.bpj.2011.12.030.

(8) Amero, C.; Schanda, P.; Dura, M. A.; Ayala, I.; Marion, D.; Franzetti, B.; Brutscher, B.; Boisbouvier, J. Fast two-dimensional NMR spectroscopy of high molecular weight protein assemblies. *J Am Chem Soc* **2009**, *131* (10), 3448-3449. DOI: 10.1021/ja809880p From NLM Medline.

(9) Suloway, C.; Pulokas, J.; Fellmann, D.; Cheng, A.; Guerra, F.; Quispe, J.; Stagg, S.; Potter, C. S.; Carragher, B. Automated molecular microscopy: the new Leginon system. *J Struct Biol* **2005**, *151* (1), 41-60. DOI: 10.1016/j.jsb.2005.03.010 From NLM Medline.

(10) Zheng, S. Q.; Palovcak, E.; Armache, J. P.; Verba, K. A.; Cheng, Y.; Agard, D. A. MotionCor2: anisotropic correction of beam-induced motion for improved cryo-electron microscopy. *Nat Methods* **2017**, *14* (4), 331-332. DOI: 10.1038/nmeth.4193 From NLM Medline.

(11) Punjani, A.; Rubinstein, J. L.; Fleet, D. J.; Brubaker, M. A. cryoSPARC: algorithms for rapid unsupervised cryo-EM structure determination. *Nat Methods* **2017**, *14* (3), 290-296. DOI: 10.1038/nmeth.4169 From NLM Medline.

(12) Rohou, A.; Grigorieff, N. CTFFIND4: Fast and accurate defocus estimation from electron micrographs. *J Struct Biol* **2015**, *192* (2), 216-221. DOI: 10.1016/j.jsb.2015.08.008 From NLM Medline.

(13) Punjani, A.; Zhang, H.; Fleet, D. J. Non-uniform refinement: adaptive regularization improves single-particle cryo-EM reconstruction. *Nat Methods* **2020**, *17* (12), 1214-1221. DOI: 10.1038/s41592-020-00990-8 From NLM Medline.

(14) Abramson, J.; Adler, J.; Dunger, J.; Evans, R.; Green, T.; Pritzel, A.; Ronneberger, O.; Willmore, L.; Ballard, A. J.; Bambrick, J.; et al. Accurate structure prediction of biomolecular interactions with AlphaFold 3. *Nature* **2024**, *630* (8016), 493-500. DOI: 10.1038/s41586-024-07487-w From NLM Medline.

(15) Pettersen, E. F.; Goddard, T. D.; Huang, C. C.; Meng, E. C.; Couch, G. S.; Croll, T. I.; Morris, J. H.; Ferrin, T. E. UCSF ChimeraX: Structure visualization for researchers, educators, and developers. *Protein Sci* **2021**, *30* (1), 70-82. DOI: 10.1002/pro.3943 From NLM Medline.

(16) Guo, Y.; Feinberg, H.; Conroy, E.; Mitchell, D. A.; Alvarez, R.; Blixt, O.; Taylor, M. E.; Weis, W. I.; Drickamer, K. Structural basis for distinct ligand-binding and targeting properties of the receptors DC-SIGN and DC-SIGNR. *Nature Structural & Molecular Biology* **2004**, *11* (7), 591-598. DOI: 10.1038/nsmb784 From www.nature.com.

(17) Tian, C.; Kasavajhala, K.; Belfon, K. A. A.; Raguette, L.; Huang, H.; Migues, A. N.; Bickel, J.; Wang, Y.; Pincay, J.; Wu, Q.; et al. ff19SB: Amino-Acid-Specific Protein Backbone Parameters Trained against Quantum Mechanics Energy Surfaces in Solution. *J Chem Theory Comput* **2020**, *16* (1), 528-552. DOI: 10.1021/acs.jctc.9b00591 From NLM Medline.

(18) Lopez, E. D.; Arcon, J. P.; Gauto, D. F.; Petruk, A. A.; Modenutti, C. P.; Dumas, V. G.; Marti, M. A.; Turjanski, A. G. WATCLUST: a tool for improving the design of drugs based on protein-

water interactions. *Bioinformatics* **2015**, *31* (22), 3697-3699. DOI: 10.1093/bioinformatics/btv411 From NLM Medline.

(19) Kozakov, D.; Grove, L. E.; Hall, D. R.; Bohnuud, T.; Mottarella, S. E.; Luo, L.; Xia, B.; Beglov, D.; Vajda, S. The FTMap family of web servers for determining and characterizing ligand-binding hot spots of proteins. *Nat Protoc* **2015**, *10* (5), 733-755. DOI: 10.1038/nprot.2015.043.

(20) Feinberg, H.; Mitchell, D. A.; Drickamer, K.; Weis, W. I. Structural Basis for Selective Recognition of Oligosaccharides by DC-SIGN and DC-SIGNR. *Science* **2001**, *294* (5549), 2163-2166. DOI: 10.1126/science.1066371.

(21) Abraham, M. J.; Murtola, T.; Schulz, R.; Páll, S.; Smith, J. C.; Hess, B.; Lindahl, E. GROMACS: High performance molecular simulations through multi-level parallelism from laptops to supercomputers. *SoftwareX* **2015**, *1-2*, 19-25. DOI: https://doi.org/10.1016/j.softx.2015.06.001.

(22) Lindorff-Larsen, K.; Piana, S.; Palmo, K.; Maragakis, P.; Klepeis, J. L.; Dror, R. O.; Shaw, D. E. Improved side-chain torsion potentials for the Amber ff99SB protein force field. *Proteins* **2010**, *78* (8), 1950-1958. DOI: 10.1002/prot.22711 From NLM Medline.

(23) Jorgensen, W. L.; Chandrasekhar, J.; Madura, J. D.; Impey, R. W.; Klein, M. L. Comparison of simple potential functions for simulating liquid water. *The Journal of Chemical Physics* **1983**, *79* (2), 926-935. DOI: 10.1063/1.445869 (acccessed 7/29/2025).

(24) McGibbon, R. T.; Beauchamp, K. A.; Harrigan, M. P.; Klein, C.; Swails, J. M.; Hernandez, C. X.; Schwantes, C. R.; Wang, L. P.; Lane, T. J.; Pande, V. S. MDTraj: A Modern Open Library

for the Analysis of Molecular Dynamics Trajectories. *Biophys J* **2015**, *109* (8), 1528-1532. DOI: 10.1016/j.bpj.2015.08.015 From NLM Medline.

(25) Kapp-Joswig, J.-O. *Normalised mutual information analysis (Jupyter notebook)*. 2025. https://github.com/janjoswig/shiny-md-collection/blob/main/shiny/procedures/mutual_information/nmi.ipynb (accessed 2025.

(26) Katoh, K.; Rozewicki, J.; Yamada, K. D. MAFFT online service: multiple sequence alignment, interactive sequence choice and visualization. *Brief Bioinform* **2019**, *20* (4), 1160-1166. DOI: 10.1093/bib/bbx108 From NLM Medline.

(27) Crooks, G. E.; Hon, G.; Chandonia, J. M.; Brenner, S. E. WebLogo: a sequence logo generator. *Genome Res* **2004**, *14* (6), 1188-1190. DOI: 10.1101/gr.849004 From NLM Medline.

(28) Zelensky, A. N.; Gready, J. E. The C-type lectin-like domain superfamily. *Febs J* **2005**, *272* (24), 6179-6217. DOI: 10.1111/j.1742-4658.2005.05031.x.
